# Delivery, Persistence, and Functional Response of dsRNA in *Acyrthosiphon pisum*: Progress and Remaining Barriers

**DOI:** 10.1002/arch.70210

**Published:** 2026-09-14

**Authors:** Lisa Merkel, Mohammad Vatanparast, Torsten Will, Silvio Erler, Khalid Amari

**Affiliations:** ^1^ Julius Kühn Institute (JKI) ‐ Federal Research Centre for Cultivated Plants Institute for Biosafety in Plant Biotechnology Quedlinburg Germany; ^2^ Zoological Institute Braunschweig Germany; ^3^ Julius Kühn Institute (JKI) ‐ Federal Research Centre for Cultivated Plants Institute for Resistance Research and Stress Tolerance Quedlinburg Germany; ^4^ Julius Kühn Institute (JKI) ‐ Federal Research Centre for Cultivated Plants Institute for Bee Protection Braunschweig Germany

## Abstract

Aphids are major agricultural pests due to their rapid reproduction, insecticide resistance, and role as vectors of plant pathogens. RNA interference (RNAi) has emerged as a promising strategy for species‐specific pest control. However, RNAi in aphids remains inconsistent, limited by dsRNA instability, inefficient uptake, and variable gene silencing. Here, we investigate key biological barriers limiting RNAi efficiency by evaluating the delivery, stability, and efficacy of double‐stranded (ds)RNA in the pea aphid *Acyrthosiphon pisum*. Feeding bioassays using food dyes and Cy3‐labeled dsRNA demonstrated ingestion and persistence of fluorescent signals within the aphid midgut for up to 120 h, confirming that the feeding system effectively delivers dsRNA to the insects. Ex vivo degradation assays revealed rapid dsRNA breakdown by aphid nucleases, with complete degradation within 2 h. Formulations improved stability: chitosan‐protected dsRNA resisted aphid nuclease activity, and layered double hydroxide (LDH) nanosheets induced elevated expression of RNAi‐associated genes. Functional assays targeting the *vacuolar H* ^*+*^ *‐ATPase 26 kDa‐like* gene (*apvhal26*) produced variable knockdown but consistently induced the expression of RNAi‐associated genes (*ago2, dicer1*), suggesting activation of the RNAi pathway or recognition of exogenous dsRNA despite inconsistent gene silencing. Our results demonstrate that several key barriers to RNAi in aphids can be partially mitigated through improved delivery and nanoparticle formulations. However, rapid nuclease‐mediated degradation and inconsistent target gene silencing remain major obstacles to reliable RNAi efficacy. This study highlights both the potential and limitations of RNAi for aphid control and emphasizes the need for optimized formulations, target gene selection, and delivery methods to advance RNAi as a sustainable biopesticide strategy.

## Highlights

- dsRNA is efficiently ingested by *Acyrthosiphon pisum* and remains detectable in the midgut.
- Aphid‐derived nucleases rapidly degrade unprotected dsRNA.
- Chitosan and LDH formulations improve dsRNA stability and enhance RNAi activation.
- RNAi machinery is activated despite variable target gene knockdown.

## Graphical abstract

Aphids efficiently ingest dsRNA, which persists in the midgut but is rapidly degraded by aphid nucleases when unprotected. Chitosan and LDH‐based formulations improve dsRNA stability, triggering RNAi activation. Despite this protection, gene knockdown remains variable, though the RNAi machinery is consistently activated.

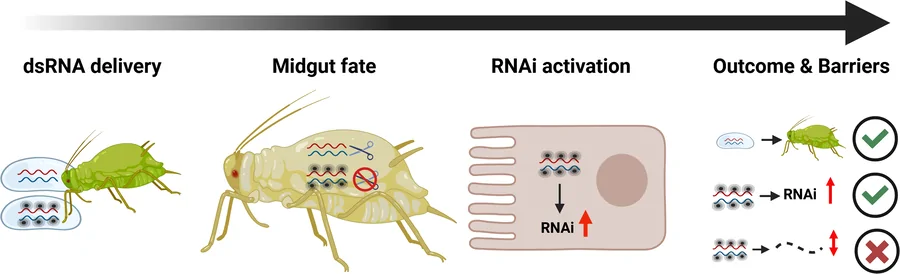

## 1 Introduction

Aphids are among the most damaging agricultural pests, responsible for substantial global yield losses through feeding on crop plants and their ability to transmit plant pathogens (Omkar 2020; Cheng et al. 2024). With nearly 4000 species colonizing about a quarter of known plants (Dixon et al. 1987), they are particularly problematic in temperate regions. In Europe, aphid infestations are estimated to cause annual losses of 700,000 tons of wheat, 850,000 tons of potatoes, and 2 million tons of sugar beet (Dedryver et al. 2010). Current management relies heavily on synthetic chemical insecticides applied via foliar sprays, seed coatings, or soil treatments (Akashe et al. 2018). However, intensive pesticide use has promoted insecticide resistance (Carletto et al. 2010; De Rouck et al. 2023) and pest resurgence (Abbasi et al. 2022). These concerns, along with the political will to reduce the use of conventional chemical synthetic pesticides, underscore the urgent need for effective, environmentally sustainable alternatives, as outlined in the EU's Green Deal.

First discovered in *Caenorhabditis elegans* (Fire et al. 1998), the RNA interference (RNAi) based conserved post‐transcriptional gene silencing mechanism is triggered by double‐stranded RNA (dsRNA), which Dicer processes into small RNAs that bind to Argonaute proteins to guide sequence‐specific degradation of target messenger RNAs (mRNA) (Jain et al. 2021). Eukaryotic RNAi pathways are highly conserved and contribute to critical biological processes (e.g., antiviral defense, gene expression control, and genome integrity) (Christiaens et al. 2020). Its high sequence specificity enables target pest control with minimal off‐target effects on non‐target organisms (NTOs), offering clear advantages over conventional insecticides (Christiaens and Smagghe 2014).

Efficacy has been demonstrated in various insect orders, particularly Coleoptera (Liu et al. 2020; Price and Gatehouse 2008). RNAi has emerged as a promising strategy for crop protection, and the first RNAi‐based commercial insecticide, Calantha, developed by Greenlight Bioscience, targets the Colorado potato beetle via dsRNA‐mediated silencing of PSMB5 (Rodrigues et al. 2021; Pallis et al. 2023).

Since the early 2010s, RNAi has been proposed as a promising, species‐specific tool for insect pest management; however, its practical implementation in aphids and other hemipterans has proven challenging, with gene silencing outcomes often remaining variable and difficult to reproduce (Christiaens and Smagghe 2014; Palli 2023; Jain et al. 2021). The possibilities for oral application to aphids are challenging due to their feeding strategy, for example, the consumption of sieve element sap from sieve tubes, which are located deep within plant tissue. This requires uptake of externally applied dsRNA sprayed onto the plant surface and its distribution within the plant, similar to systemic insecticides. Once ingested, dsRNA was reported to be rapidly degraded by salivary and gut nucleases, limiting its bioavailability (Christiaens and Smagghe 2014; Elston et al. 2023). For example, RNAi against the salivary effector gene *C002* induced high mortality rates when injected into *Acyrthosiphon pisum* (Mutti et al. 2008), but showed minimal effects when delivered via feeding or transgenic plants (Pitino et al. 2011; Pitino and Hogenhout 2013). In contrast, the transgenic production of dsRNA targeting *shp* in the aphid *Sitobion avenae* resulted in strong direct and multigenerational silencing effects (Abdellatef et al. 2015). Similarly, RNAi‐mediated knockdown of the *ecdysone receptor* (*EcR*), which regulates wing development, has been demonstrated in pea aphids following dsRNA injection, although the magnitude of phenotypic effects was variable. While informative mechanistically, injection‐based delivery represents a highly invasive approach that is not directly comparable to oral or topical RNAi strategies relevant for field applications (Vellichirammal et al. 2017). These challenges include, for both plants and insects, limited cellular uptake, inefficient systemic RNAi, and rapid degradation of dsRNA.

To overcome these barriers, nanoparticle‐based formulations have been developed to protect dsRNA and facilitate uptake. Chitosan, a cationic polysaccharide, can electrostatically bind dsRNA into nanoparticles that shield it from nucleases, enhance stability inside the insect gut, and facilitate uptake by insect cells of the alimentary tract (Yang et al. 2022; Zhou et al. 2023; Vatanparast and Amari 2025; Vatanparast et al. 2024; Almasi and Amari 2021). Chitosan‐based formulations have improved RNAi efficacy in several insect species, including mites and locusts (Kolge et al. 2021; Liu et al. 2025b), and are biodegradable, inexpensive, and environmentally safe. Another formulation uses layered double hydroxide (LDH) nanosheets, also known as “BioClay.” These positively charged mineral layers adsorb dsRNA and slowly release it, providing extended protection against environmental degradation and improved delivery to sap‐feeding insects (Cheng et al. 2024; Mitter et al. 2017b). LDH has been shown to maintain dsRNA integrity on leaf surfaces for weeks and enhances RNAi efficiency across multiple pest species (Jain et al. 2022).

The *vacuolar H*+*‐ATPase 26 kDa‐like* gene (*apvhal26*) was chosen as the primary target gene for this study. In *A. pisum, apvhal26* encodes a component of the V1 sector of the V‐ATPase proton pump, a highly conserved multi‐subunit complex that uses ATP hydrolysis to energize proton transport, thereby acidifying intracellular compartments, which is essential for pH homeostasis, vesicular trafficking, endocytosis, and ion transport in eukaryotic cells (Forgac 2007; Niu et al. 2019). V‐ATPase subunits are broadly expressed across insect tissues involved in membrane transport (e.g., gut epithelia, Malpighian tubules, salivary glands), fulfilling vital roles in nutrient absorption, osmoregulation, excretion, and survival. Based on knowledge of variable responses based on the target gene, a secondary target gene, *macrophage migration inhibitory factor 1* (*MIF1*), was chosen, a salivary effector protein that is secreted during feeding and plays a key role in host plant immune suppression, thereby enabling successful host colonization (Liu et al. 2021; Naessens et al. 2015). Both target genes are highly conserved across aphid species.

This study investigated the biological factors influencing RNAi efficiency in the pea aphid *A. pisum*, with a particular focus on dsRNA stability, delivery, and formulation strategies designed to improve RNAi performance. Oral feeding and topical application were evaluated as dsRNA delivery routes, and nanoparticle formulations based on chitosan and LDH were assessed for their potential to improve dsRNA stability and biological response. By systematically comparing delivery routes, target genes, and dsRNA formulations, this study aims to improve our understanding of the biological factors influencing RNAi efficacy in aphids and to identify where major barriers to reproducible gene silencing remain.

## 2 Materials and Methods

### 2.1 Aphid Rearing and Diet

*A. pisum* was originally collected from *Vicia faba* (broad bean) plants in the field near Quedlinburg, Germany (51°46′21.6″N 11°08′52.0″E). Colonies were maintained in a controlled climatic chamber under long‐day conditions (16 h light:8 h dark) at 20°C–22°C and 70 ± 5% relative humidity. Aphids were reared on healthy *V. faba* plants in pesticide‐free soil.

For all feeding experiments, an artificial diet was prepared according to a modified protocol based on Cherqui and Tjallingii (2000), as optimized by Will et al. (2008). The diet consisted of 438 mM sucrose (15% *w/v*), 100 mM l‐serine, 100 mM l‐methionine, and 100 mM l‐aspartic acid, with the pH adjusted to 7.2 using KOH. The solution was sterile‐filtered through a 0.45 μm PVDF membrane filter (Rotilabo, Carl Roth, Karlsruhe, Germany) and stored in 25 mL aliquots at –20°C until use. All procedures were performed under sterile conditions to prevent microbial contamination.

### 2.2 Bioinformatic Studies of Target Genes

Primer sequences (Table 1) and the dsRNA synthesis protocol for ds*Apvhal26* were adapted from Niu et al. (2019).

**Table 1:** Primers used for in vitro dsRNA synthesis with *T7 promoter*.

Gene	Primer direction	Sequence (5′ → 3′)	Amplicon size (bp)	Reference
*GFP*	Forward	TAATACGACTCACTATAGGGGTCAAGTTTGAGGGAGACACCC	250	This study
	Reverse	TAATACGACTCACTATAGGGAAAGGACAGGGCCATCGC		
*apvhal26*	Forward	TAATACGACTCACTATAGGGACATTGAAAAGGGCCGCCT	481	Niu et al. 2019
	Reverse	TAATACGACTCACTATAGGGGCGATTCCAGTGTGTTACAAAT		
*MIF1*	Forward	TAATACGACTCACTATAGGGAATATGCCTCATTTCCGTTTGG	223	This study
	Reverse	TAATACGACTCACTATAGGGTGTTCTGTTCAACGCCTAGG		

*Note:* Sequences of primers for the synthesis of DNA templates to generate dsRNA via in vitro transcription.

To ensure that the designed dsRNA would be efficient across multiple aphid species, the *A. pisum MIF1* coding sequence was aligned to *Myzus persicae* using the BLAST nucleotide alignment tool (NCBI, https://blast.ncbi.nlm.nih.gov). Conserved regions between the two species were identified, and a 223‐nucleotide target sequence within the *A. pisum MIF1* gene was selected for dsRNA synthesis. Candidate regions were initially evaluated using the E‐RNAi webserver (currently offline), which allowed prediction of potential off‐target effects and provided guidance for effective siRNA segment design.

To enable both gene expression analysis and in vitro transcription of dsRNA from a single primer pair, all primers were synthesized with a T7 RNA polymerase promoter sequence (5′‐TAATACGACTCACTATAGGG‐3′) appended to the 5′ end (Table 1).

### 2.3 In Vitro Transcription of dsRNA

The dsRNAs were designed to target *apvhal26* and *MIF1*, which play important roles in the survival of *A. pisum* and *M. persicae*. DsRNA for the *green fluorescent protein* (*GFP*) was used as a control. Total RNA was extracted from ten 5‐day‐old adult aphids using TRIzol Reagent and Direct‐zol RNA Miniprep Plus (ZYMO Research, Freiburg, Germany) and then prepared for cDNA synthesis using Moloney Murine Leukemia Virus Reverse Transcriptase (M‐MLV RT) (Promega, Madison, WI, USA). All primers were synthesized by Metabion (Metabion International AG, Steinkirchen, Germany). The target gene sequence fragments were amplified with Taq polymerase and used as templates for dsRNA synthesis with a T7 RiboMAX Express RNAi System (Promega, Madison, WI, USA) according to the manufacturer's protocol. DsRNA targeting *GFP* was used as a non‐target insect‐specific control.

### 2.4 Preparation of Formulation and dsRNA/Formulation Nanoparticles

**Figure 1:**
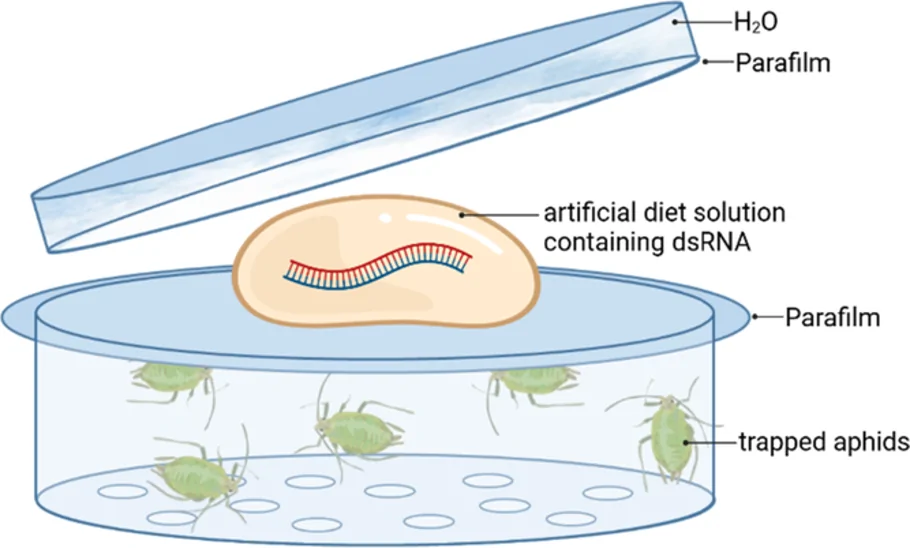
Schematic of the *Acyrthosiphon pisum* feeding chamber for dsRNA delivery. Feeding chamber constructed from 35 mm Petri dishes containing five *A. pisum* adults (3 days old). UV‐sterilized Parafilm encloses aphids in the chamber. Positioned on top, 100 μL of artificial aphid diet containing 250 ng/μL dsRNA was added for aphid ingestion. A lid filled with nuclease‐free water provided gentle pressure to maintain the feeding setup. *Source:* Figure created on September 23, 2025, with https://www.biorender.com/.

Chitosan/dsRNA nanoparticles were synthesized following a modified protocol adapted from Zhang et al. (2010) and Vatanparast and Amari (2025) (see Supporting Information S1: Figure 1).

**Figure 2:**
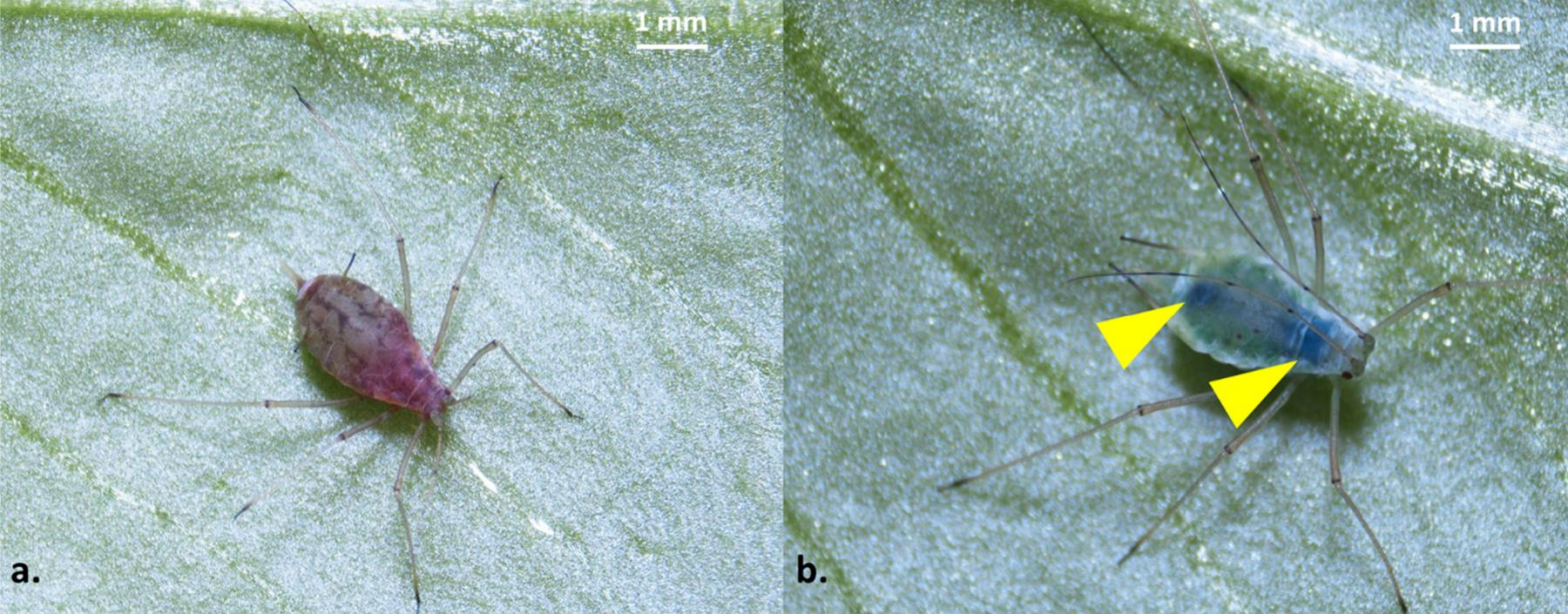
Visual inspection of food dye‐fed aphids. (a) *Acyrthosiphon pisum* adult exhibiting strong red staining after 72 h of feeding on an artificial diet containing 0.1% Allura Red AC. (b) *A. pisum* adult displaying internal blue coloration following ingestion of an artificial diet containing 0.1% Brilliant Blue FCF. Dye accumulates in the stomach and hindgut (indicated by yellow arrows).

MgAl‐layered double hydroxide (MgAl‐LDH) nanoparticles were synthesized following a modified protocol adapted from previous studies (Dong et al. 2014; Mitter et al. 2017a; Xu et al. 2006) (see Supporting Information S1: Figure 2).

### 2.5 Uptake of dsRNA Into Aphids

#### 2.5.1 Food Dyes

To verify successful ingestion of the artificial diet and confirm oral uptake of dsRNA by *A. pisum*, two food‐grade dyes, Brilliant Blue FCF and Allura Red AC, were used as visual markers, following the protocol established by Ehrenberg et al. (2019). Each dye was prepared as a 0.1% (*w/v*) solution in Tris‐HCl buffer, and 1 μL of the resulting solution was added to 100 μL of artificial diet, prepared as described above. The stained diets were then applied to an artificial feeding chamber as described by Mitter and Dadd (1964). Feeding cages were constructed using 35‐mm‐diameter Petri dishes, with the bottom removed and replaced by perforated Parafilm M (Pechiney Plastic Packaging, Chicago, IL, USA) to allow airflow. After placing the aphids inside the cages, the Petri dishes were sealed with UV‐sterilized, cleaned 2 cm^2^ Parafilm M pieces, which were then carefully stretched to facilitate aphid penetration. Next, 100 μL of feeding mixture was applied to the Parafilm M and covered with an additional Parafilm M layer (Figure 1). Aphids were allowed to feed under identical environmental conditions for 72 h. For visual inspection, a stereomicroscope was used.

#### 2.5.2 Observation of Cy3‐Labeled dsRNA

To further confirm dsRNA uptake via ingestion, a fluorochrome‐labeled long dsRNA construct (dsGFP, 500 ng/μL) was prepared using the Silencer siRNA Labeling Kit with Cy3 dye (Thermo Fisher Scientific, Waltham, MA, USA), following the manufacturer's protocol. The labeled dsRNA was incorporated into the artificial diet (5 μg Cy3‐labeled ds*GFP*), and adult *A. pisum* were exposed to a 100 μL mixture using the same feeding chamber setup as described above. Aphids were allowed to feed on the artificial diet mixture for 48 h. Subsequently, five aphids per treatment group were transferred to a blank artificial diet. Aphids were then examined by fluorescence microscopy at 0.5, 1, 2, 24, 48, 96, and 120 h after transfer (Nikon Eclipse Ni‐E, Tokyo, Japan) equipped with a PF 4× Cy3 filter set (ZM: 1.0×, FS 30.6 mm). A control group of aphids was fed an artificial diet mixture containing only the Cy3‐labeling reagent. Additionally, chambers containing only nuclease‐free water and artificial diet served as negative controls.

### 2.6 Ex Vivo Degradation of dsRNA in *A. pisum* Whole‐Body Extracts

**Figure 3:**
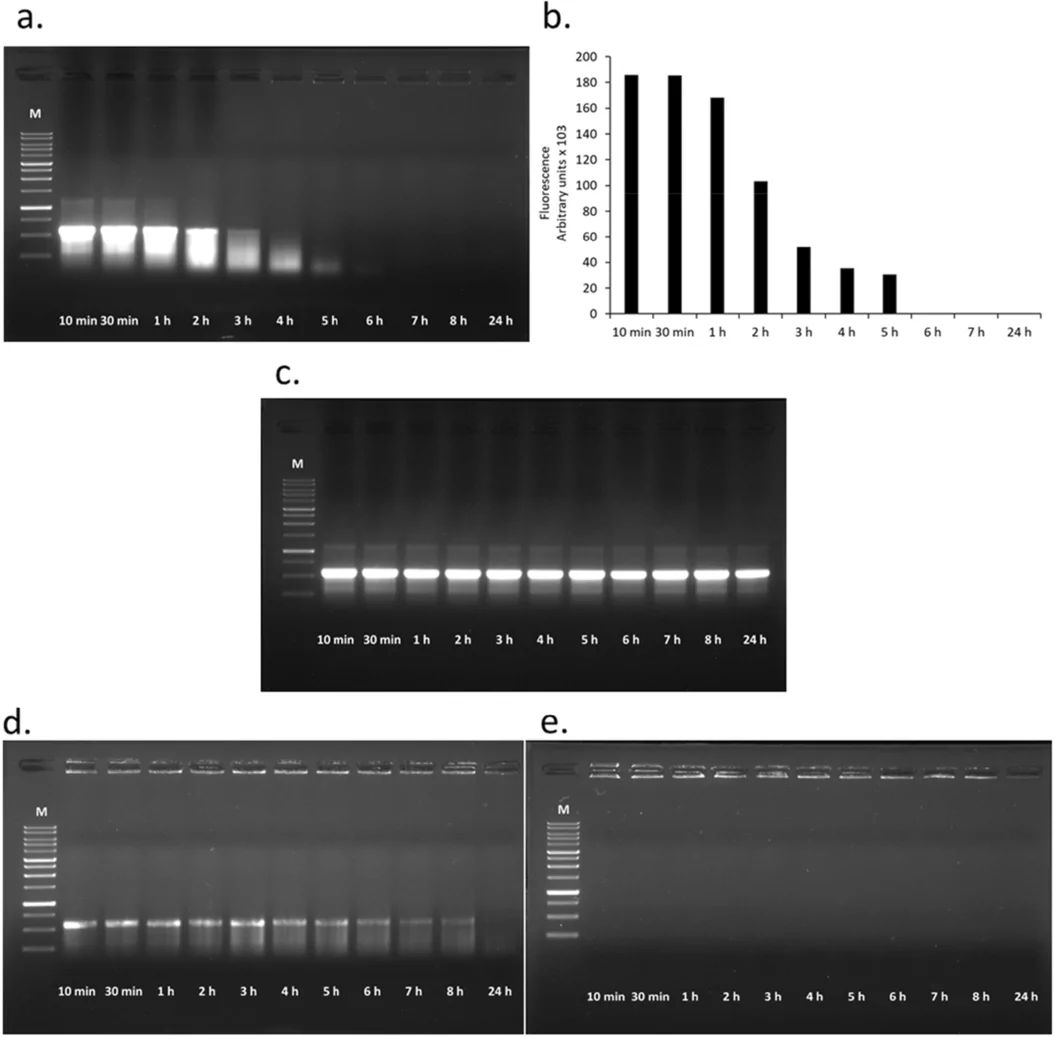
Degradation of unformulated and formulated dsRNA. (a) Agarose gel electrophoresis showing the progression of dsRNA degradation by *Acyrthosiphon pisum* BE. Four micrograms of ds*Apvhal26* were added to a BE solution containing 10 μg of total protein for up to 24 h. (b) Fluorescence intensity (arbitrary units) of dsRNA bands in agarose gel in (a). Fluorescence intensity was quantified using ImageJ software. (c) Stability of unformulated ds*Apvhal26* incubated at room temperature without *A. pisum* BE for 24 h. No signal weakening was detectable over the course of 24 h. (d) Stability of chitosan‐formulated ds*Apvhal26* after incubation in *A. pisum* BE over time. Due to electrostatic interactions with chitosan, the dsRNA remains bound in nanoparticle complexes and does not migrate through the gel, instead accumulating in the wells. Band intensity within the wells was used to assess stability. A strong signal was retained in the well for up to 5 h, with slight weakening thereafter, but a detectable signal persisted throughout the entire time course, indicating effective protection from nuclease‐mediated degradation. Limited release of free dsRNA from the complexes was observed during incubation, with this released fraction also persisting longer than unformulated dsRNA. A weak but detectable fluorescent signal occurs in the well at 24 h of incubation. (e) Stability of LDH‐formulated ds*Apvhal26* after incubation in *A. pisum* BE over time. Nanoparticles do not migrate through the gel; instead, they accumulate in the wells. Band intensity within the wells was used to assess stability. A strong signal persisted for up to 8 h, with slight weakening thereafter, and a detectable signal persisted throughout the entire time course, indicating effective protection from nuclease‐mediated degradation. After 24 h of incubation, no fluorescent signal was detected. “M” indicates the DNA size marker (1 kb ladder).

To assess the stability of in vitro transcribed ds*GFP* and ds*Apvhal26*, degradation assays were performed using whole‐body extracts (BE) from *A. pisum*. Fifty 3‐day‐old adult aphids were collected into a 1.5 mL microcentrifuge tube containing 100 μL of 1× phosphate‐buffered saline (PBS), corresponding to 2 μL PBS per aphid. For PBS, a pH of 7.0 was chosen to investigate the potential influence of RNA‐degrading enzymes during transfer through cells, including the intestinal epithelium, as this represents the mean pH of the midgut region (Cristofoletti et al. 2003), the gut region with significant endonuclease activity (Ghodke et al. 2019). Aphids were homogenized manually using disposable pestles (Eppendorf micropestles, Hamburg, Germany). The homogenate was centrifuged at 12,000×*g* for 20 min at 4°C to pellet tissue debris. Following centrifugation, the upper fat‐free phase of the two‐layered supernatant was carefully collected and subjected to a second centrifugation under identical conditions to remove residual particulates. The resulting clarified body extract was collected and stored on ice for subsequent use in degradation assays after determining the total protein concentration. The total protein concentration of the aphid BE was quantified using the Pierce BCA Protein Assay Kit (Thermo Fisher Scientific, Waltham, MA, USA) according to the manufacturer's instructions. Protein concentrations were determined by comparison to a standard curve generated using bovine serum albumin (BSA) as a reference (Supporting Information S1: Figure 3).

For the degradation experiments, 4 μg of unformulated ds*Apvhal26*, 4 μg of chitosan‐bound ds*Apvhal26* (dsRNA‐to‐chitosan ratio 1:4), as well as 4 µg of LDH‐bound ds*Apvhal26* (dsRNA‐to‐LDH ratio 1:7) were incubated at room temperature with 1 μL BE containing 10 μg of total protein. Additionally, unformulated ds*Apvhal26* without BE was incubated at room temperature as a control. Samples were collected and immediately frozen after 1 min, 30 min, 1, 2, 3, 4, 5, 6, 7, 8, and 24 h and later analyzed on a 1% agarose gel. Fluorescence intensity of the gel bands was quantified using ImageJ software (1.54 g).

### 2.7 Methodology of Different dsRNA Application Methods

#### 2.7.1 Feeding Bioassay—Oral dsRNA Delivery

Bioassay studies were conducted to determine the effect of dsRNA on both target genes. In feeding experiments, *A. pisum* adults were exposed to either dsRNA or dsRNA/chitosan nanoparticles. Two controls, untreated (nuclease‐free water) and non‐target dsRNA (ds*GFP*), were also employed in this study. Using a fine paintbrush, five age‐synchronized *A. pisum* adults (3–5 days after fourth nymph instar) were transferred from *V. faba* to feeding chambers as described in Section 2.5.2. Five aphids were placed into one feeding chamber, with five repetitions per treatment. The feeding mixture contained dsRNA or dsRNA/chitosan nanoparticles at a final concentration of 250 ng/μL, along with an artificial aphid diet. The artificial diet was prepared as described in Section 2.1. The feeding cages were placed in a climate chamber (60% relative humidity; 2°C during the day, 18°C at night; 15.043 lux), programmed for a 16‐h light/8‐h dark cycle, with light intensity set to 60%. The aphids remained undisturbed in the feeding chambers for 72 h. After a subsequent 72 h resting period on *V. faba* leaves, aphids were collected for target gene transcription level analysis. In each treatment (ds*GFP*, ds*Apvhal26*, water), 6–8 aphids were pooled into a single biological replicate for RNA extraction and subsequent qPCR analysis (see Section 2.8), and at least five biological replicates were included per treatment.

#### 2.7.2 Topical dsRNA Delivery

The topical dsRNA delivery was administered according to Niu et al. (2019). In short, 3–5‐day‐old *A. pisum* adults were placed on double‐sided tape to immobilize the aphids. 1 μL of a solution containing 540 ng of dsRNA (*GFP, Apvhal26*) or a control solution (nuclease‐free water) was placed on the dorsal side of the aphid abdomen. After a 30‐min inoculation period, the solution was completely dry, and the aphids were removed from the tape by placing a small amount of nuclease‐free water around them to loosen the tape adhesive. After 36 h of exposure, the aphids were placed back on their host plant seedling (*V. faba*) for a subsequent 72 h resting period, as described by Niu et al. (2019). After the resting period on *V. faba* leaves, aphids were collected for target gene expression analysis. Samples were collected in the same way as described in Section 2.7.1.

### 2.8 Quantitative Real‐Time PCR (qRT‐PCR)

To assess whether dsRNA exposure triggered gene silencing in *A. pisum*, qPCR was performed to measure target gene transcript levels following treatment. Aphids were collected after exposure to dsRNA, either via feeding or topical application, and immediately preserved in 250 μL of TRIzol Reagent (Invitrogen, Thermo Fisher Scientific). Each sample consisted of 6–8 aphids in 1.5 mL microcentrifuge tubes. An additional 550 μL of TRIzol was added, and the samples were thoroughly homogenized using disposable plastic pestles. Total RNA was extracted using the Direct‐zol RNA Miniprep Kit (Zymo Research, Irvine, CA, USA) according to the manufacturer's protocol, including on‐column DNase treatment. Total RNA (1 µg) was reverse transcribed using an oligo(dT) primer. First‐strand cDNA synthesis and subsequent qPCR were carried out using the GoTaq 2‐Step RT‐qPCR System (Promega, Madison, WI, USA) and a qTOWER^3^ G thermocycler (Analytik Jena). Gene expression levels were normalized to the endogenous reference gene *succinate dehydrogenase complex subunit B* (*SDHB*) (Yang et al. 2014), which served as an internal control for cDNA input.

In addition to evaluating potential downregulation of the dsRNA‐specific target genes, the expression of two core components of the RNA interference pathway, *argonaute* (*ago2*) and *dicer* (*dicer1*), was also examined. Since exogenous dsRNA is expected to activate the RNAi machinery, transcript levels of *ago2* and *dicer1* were quantified to assess molecular activation of the silencing response. For primer sequences used for qPCR, see the supplemental material (Table S1). Relative gene expression levels were calculated using the 2^−ΔΔ*C*^ *t* method (Livak and Schmittgen 2001). For each sample, the threshold cycle (*C_t_*) value of the target gene (*vacuolar H* ^*+*^ *‐ATPase*) was first normalized to the *C_t_* value of the internal reference gene *SDHB* to obtain the ΔC_t_ value. The resulting Δ*C_t_* values from treatment groups were then compared with those of the non‐targeting dsRNA (ds*GFP*)‐treated group. To allow comparison across treatments, expression values were normalized relative to the mean expression of the H_2_O‐control.

All qPCR reactions were performed in technical triplicate, and results are reported as mean ± standard error of the mean (SEM). Statistical significance of gene down‐ or upregulation between treatment and control groups was assessed using a two‐tailed Student's *t* test, with *p* values < 0.05 considered statistically significant. Statistical analysis and graph generation were performed using GraphPad Prism 11.

## 3 Results

### 3.1 Uptake of dsRNA Into *A. pisum*

#### 3.1.1 Food Dyes

Visual inspection under a stereomicroscope confirmed successful ingestion of the dyed diet in all tested aphids (Brilliant Blue FCF, *n* = 38; Allura Red AC, *n* = 36), evidenced by a clear red or blue coloration within the gut and body cavity (Figure 2). In both cases, aphids exhibited characteristic internal staining after feeding on the dyed diet. While the blue dye accumulated in the stomach and hindgut (Figure 2a, indicated by yellow arrowheads), passing the midgut, the red dye dispersed into the hemocoel, thereby providing unambiguous confirmation that the aphids had actively fed on the provided diet (Figure 2b).

#### 3.1.2 Validation of dsRNA Uptake and Localization in *A. pisum*

**Figure 4:**
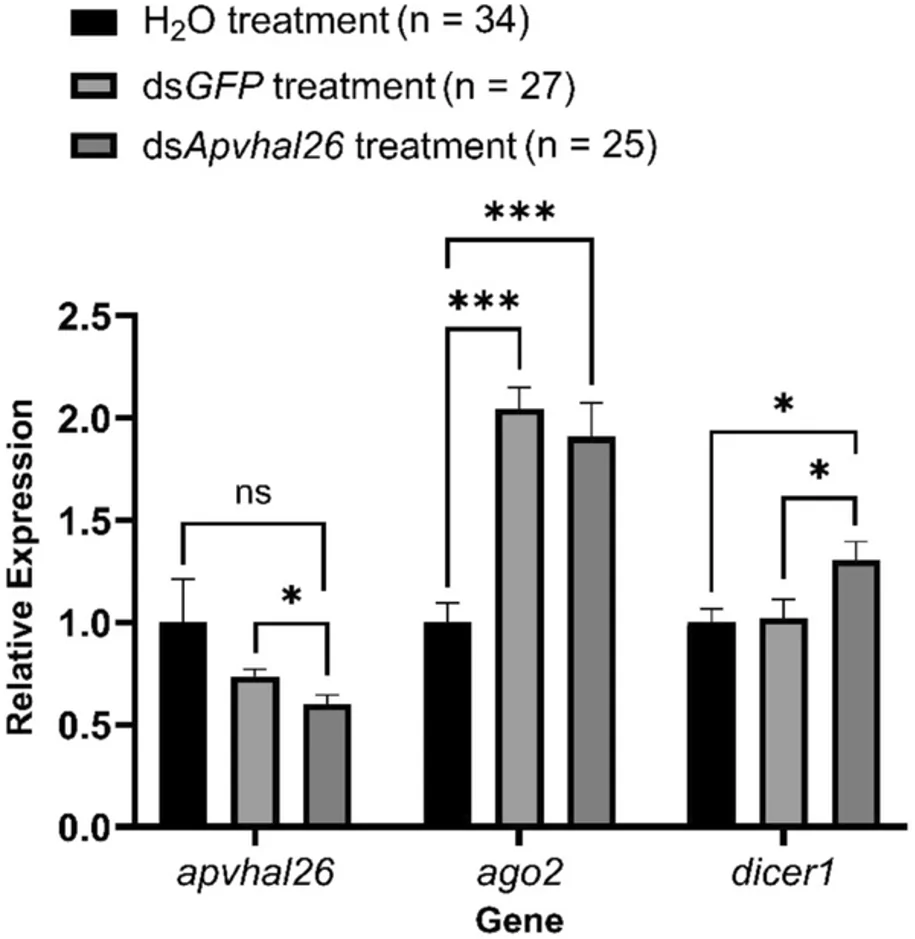
Relative gene expression of *apvhal26*, *ago2*, and *dicer1* in *Acyrthosiphon pisum* after dsRNA feeding. Relative transcript levels of *apvhal26, argonaute2* (*ago2*), and *dicer1* in aphids fed with ds*Apvhal26*, ds*GFP*, or H_2_O. Aphids fed ds*Apvhal26* showed a significant reduction in *apvhal26* expression compared to the ds*GFP* control treatment. Expression of *ago2* was significantly increased in aphids fed on ds*Apvhal26* and ds*GFP* compared to H_2_O. Relative expression of *dicer1* also showed significantly higher expression in aphids feeding on ds*Apvhal26* compared to both control groups. Bars represent normalized relative expression ± SEM. Asterisks indicate statistically significant differences between treatments (*p ≤ 0.05; **p ≤ 0.01; ***p ≤ 0.001; unpaired *t* test).

In all samples fed with ds*GFP*+Cy3, a clear Cy3 fluorescence signal was consistently detected in the midgut region at all time points, beginning as early as 30 min post‐feeding (Supporting Information S1: Figure 4a). The intensity and localization of the fluorescent signal remained largely unchanged throughout the 120 h and did not spread beyond the stomach, that is, it did not pass the intestine.

Fluorescence was also observed in the control group fed with Cy3 dye alone (without ds*GFP*), but not in the water‐control group. In both the treatment and control groups, Cy3 signal was restricted to the stomach, with no detectable fluorescence in surrounding tissues (Supporting Information S1: Figure 4b). No fluorescent signal appeared in aphids that were fed with only ds*GFP* (Supporting Information S1: Figure 4c).

#### 3.1.3 Stability of dsRNA in Ex Vivo Collected *A. pisum* Body Extract

To evaluate the susceptibility of dsRNA to degradation by aphid‐derived nucleases, unformulated and formulated ds*Apvhal26* was incubated ex vivo in *A. pisum* BE. As shown in Figures 3a,b, the unprotected dsRNA was rapidly degraded upon exposure to BE compared to dsRNA without exposure to BE (Figure 3c). Following incubation in aphid extract, degradation was already evident after 2 h. After 3 h of incubation, the full‐length dsRNA was no longer detectable, and only diffuse staining of degraded fragments remained. At the 5‐h time point, dsRNA degradation was nearly complete, with a minimal residual signal visible.

To assess the protective effect of chitosan‐based encapsulation on dsRNA stability, chitosan/ds*Apvhal26* nanoparticles were incubated under identical nuclease‐rich conditions in aphid BE. As shown in Figure 3d, chitosan‐formulated dsRNA displayed improved resistance to degradation compared to unformulated dsRNA. Because electrostatic interactions retain the chitosan‐dsRNA complexes within the loading wells during electrophoresis, stability was assessed by monitoring fluorescence intensity over time. Compared with unprotected dsRNA, chitosan‐formulated dsRNA maintained a clear fluorescent signal throughout the incubation period, indicating substantially improved resistance to nuclease‐mediated degradation. Limited release of free dsRNA from the complexes was observed during incubation, with this released fraction also persisting longer than unformulated dsRNA, showing partial degradation after 5 h and disappearance after 8 h of incubation. A slight reduction in band intensity within the gel well was also apparent after approximately 8 h, indicating partial degradation.

Comparable results were observed with dsRNA/LDH nanoparticles (Figure 3e). Degradation assays with LDH‐formulated ds*Apvhal26* showed markedly enhanced resistance to BE nuclease activity compared to unprotected dsRNA. As in the chitosan assays, LDH‐bound dsRNA remained stable throughout the incubation period, appearing as a strong, immobile band in the loading well. No release of free dsRNA from the LDH complexes was observed during the incubation period, indicating a stronger interaction between dsRNA and the LDH carrier compound compared to the chitosan formulation.

Overall, both nanoparticle formulations delayed dsRNA degradation in aphid BE relative to unformulated dsRNA, demonstrating enhanced stability under the ex vivo conditions used in this study.

### 3.2 Comparative Analysis of Gene Expression After dsRNA Treatment

#### 3.2.1 Gene Expression Analysis Following dsRNA Feeding Treatment

In a first trial, aphids treated with ds*Apvhal26* (*n* = 25) exhibited a significant reduction in *apvhal26* transcript levels compared to the ds*GFP*‐control group (*n* = 27) (Figure 4). Relative gene expression decreased by 26.2% in the ds*GFP* group compared to H_2_O, and 39.8% in the ds*Apvhal26*‐treated group compared to the H_2_O‐control group (*n* = 34) (*p* < 0.05). Transcript levels of *ago2* were significantly higher in both dsRNA‐treated groups compared to the H_2_O control (*p* < 0.001). The ds*GFP* group showed a marked increase in expression by ~100%, while the ds*Apvhal26* group exhibited a comparable but slightly lower upregulation (~90% increase).

Expression of *Dicer1* followed a similar trend, with both ds*GFP* and ds*Apvhal26* treatments leading to significantly higher transcript levels than H_2_O. At the same time, expression increased by ~2% in the ds*GFP* group and by 30.5% in the ds*Apvhal26* group (*p* < 0.05 for both comparisons with H_2_O and between ds*GFP* and ds*Apvhal26*).

**Figure 5:**
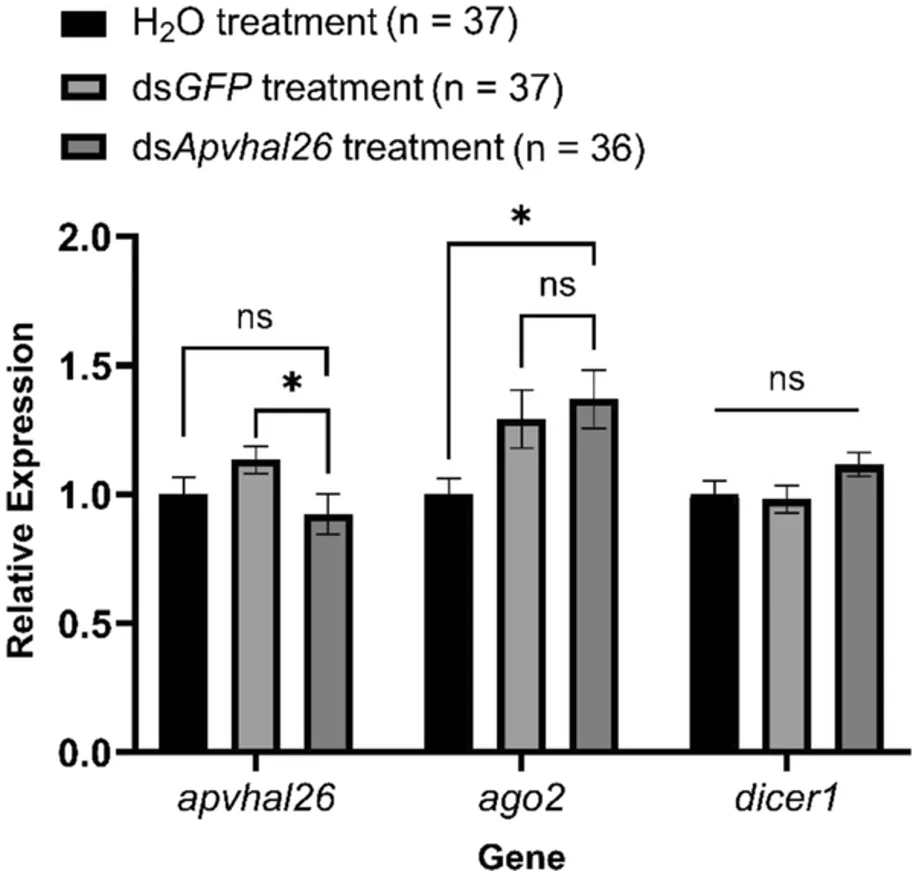
Relative expression of apvhal26, ago2, and dicer1 in *Acyrthosiphon pisum* after dsRNA feeding. Quantification of apvhal26, ago2, and dicer1 transcripts in *A. pisum*. Only apvhal26 showed downregulation for the dsApvhal26 treatment compared to dsGFP; ago2 abundance increased in dsApvhal26 compared to H_2_O. Bars represent normalized, mean relative expression ± SEM. Statistical comparisons between treatments are indicated by asterisks (*p ≤ 0.05 or “ns” not significant, unpaired *t* test).

A comparable expression pattern was observed following oral delivery of dsRNA targeting a secondary target gene, *MIF1*, with reduced target gene transcript levels (Supporting Information S1: Figure 5) accompanied by significantly increased expression of the RNAi‐associated genes *ago2* and *dicer1*.

#### 3.2.2 Gene Expression Analysis: Repetition of the dsRNA Feeding Experiment

**Figure 6:**
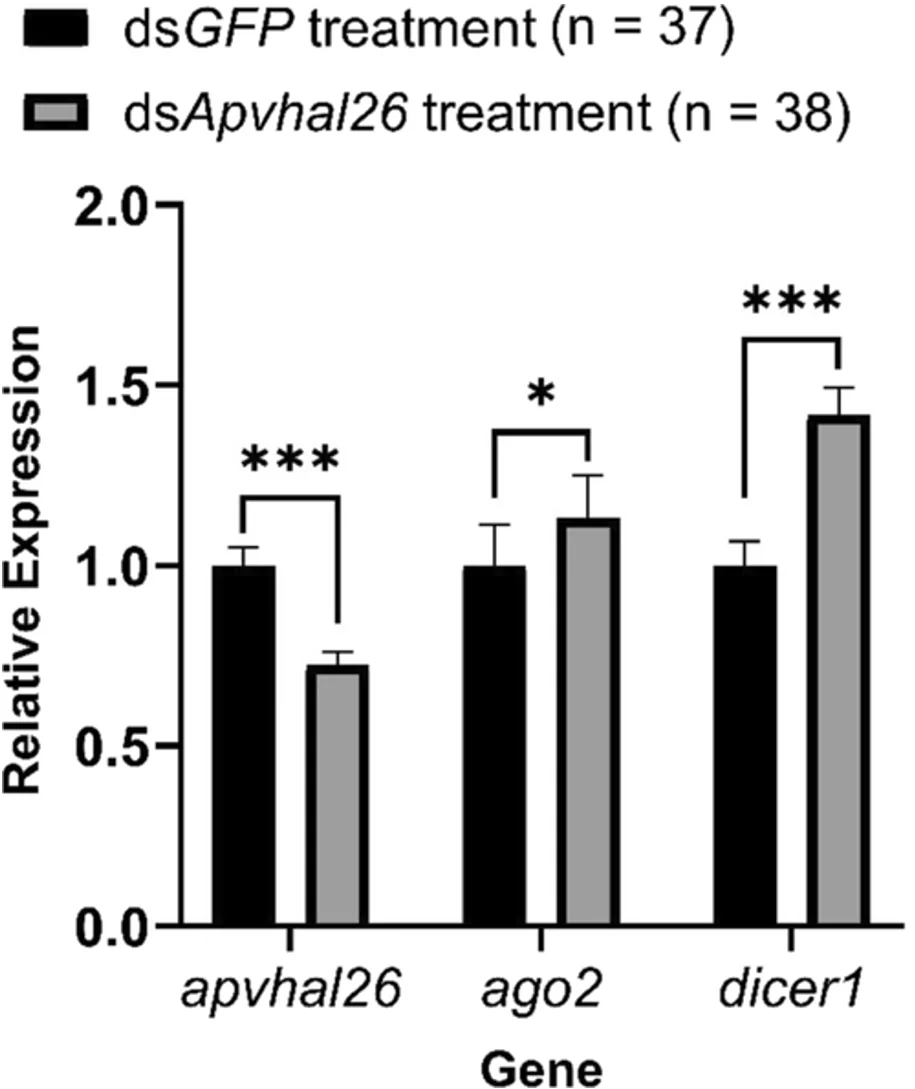
Relative expression of *apvhal2*6, *ago2*, and *dicer1* in *Acyrthosiphon pisum* after topical dsRNA delivery. Quantification of *apvhal26*, *ago2*, and *dicer1* transcripts in *A. pisum* by qRT‐PCR. The lower *apvhal26* mRNA transcript levels in aphids treated with ds*Apvhal26* showed strong statistical significance compared to the ds*GFP* control treatment. Gene expression of *argonaute2* and *dicer1* was significantly overexpressed in aphids treated with ds*Apvhal26* compared to the control group (ds*GFP*). Bars represent normalized relative expression ± SEM. Asterisks indicate statistically significant differences between treatments (*p ≤ 0.05; ***p ≤ 0.001; unpaired *t* test).

In contrast to the first experiment, the expression of *apvhal26* did not show any significant difference between the H_2_O (*n* = 37) and ds*Apvhal26* (*n* = 36) groups (Figure 5). A modest target gene reduction was observed in ds*Apvhal26*‐treated aphids compared to ds*GFP*‐fed aphids (*n* = 37) (*p* < 0.05), but transcript abundance did not differ from the H_2_O control. Expression of a*go2* was elevated in both dsRNA treatment groups compared to the water control. Although mean *ago2* expression was higher (~ 29% increase for ds*GFP* and ~37% increase for ds*Apvhal26*), differences from the H_2_O control (~ 3.1%) were not statistically significant. Unlike the previous experiment, dicer1 transcript levels showed no significant differences among the treatment groups. Expression levels remained consistent across the H_2_O, ds*GFP*, and ds*Apvhal26* treatments, with relative expression values ranging narrowly from ~0.98 to ~1.12 without statistical significance. This lack of target gene downregulation was also evident in repeated feeding experiments with oral delivery of dsRNA targeting *MIF1* (Supporting Information S1: Figure 6).

#### 3.2.3 Gene Expression Analysis Following Topical Application

**Figure 7:**
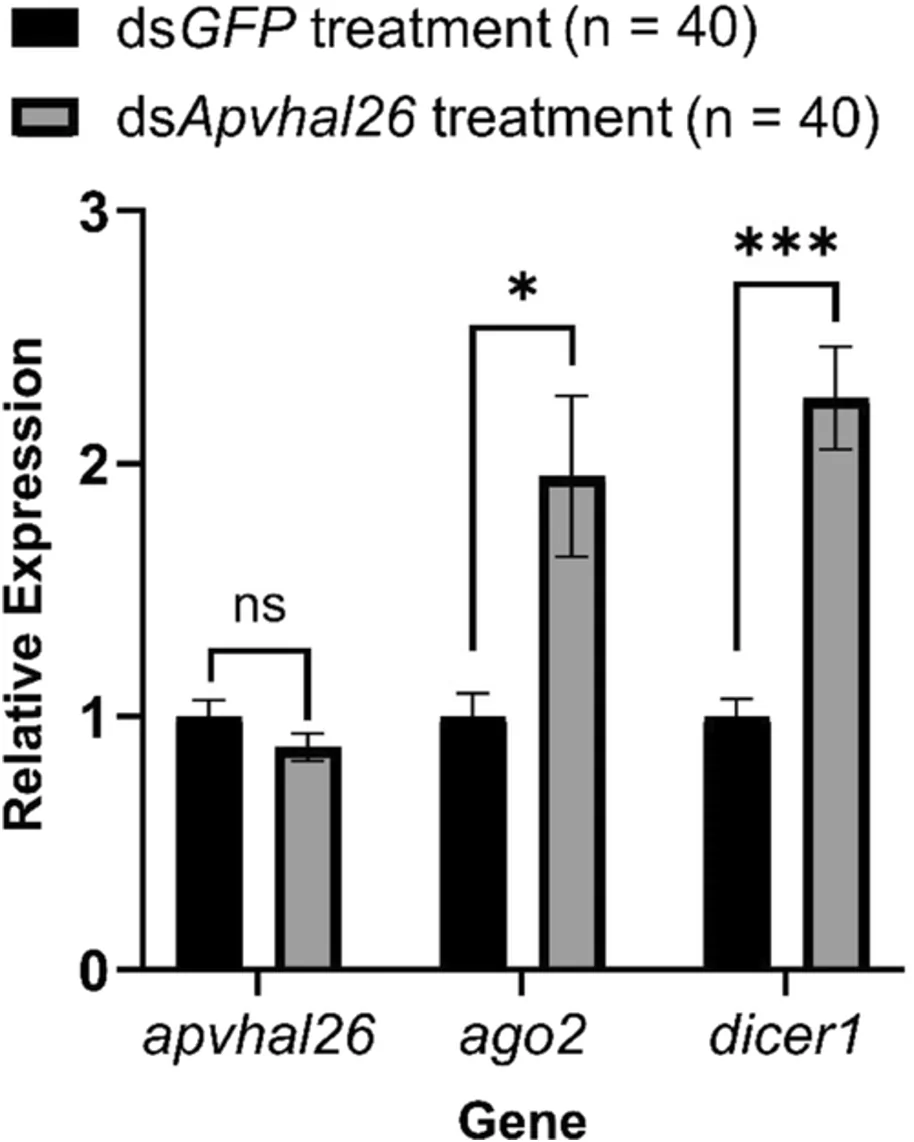
Inconsistent results of relative expression of *apvhal26*, *ago2*, and *dicer1* in *Acyrthosiphon pisum* after topical dsRNA delivery. Quantification of *apvhal26*, *ago2*, and *dicer1* transcripts in *A. pisum* by qRT‐PCR. The lower *apvhal26* mRNA transcript levels in aphids treated with ds*Apvhal26* were not statistically significant compared to the control treatment. Expression of *argonaute2* and *dicer1* was significantly higher in aphids treated with ds*Apvhal26* compared to the control group (ds*GFP*). Bars represent normalized relative expression ± SEM. Asterisks indicate statistically significant differences between treatments (*p ≤ 0.05; **p ≤ 0.01; ***p ≤ 0.001; “ns” not significant; unpaired *t* test).

In the first experiment (Figure 6), topical application of ds*Apvhal26* resulted in a significant downregulation of the target gene (*n* = 37) compared to the *GFP* control group (*n* = 38). Expression of *ago2* and *dicer1* was significantly higher in aphids treated with ds*Apvhal26* than in the ds*GFP* control. In repeated experiments using identical treatment conditions, the expression profile was less consistent (Figure 7), and the previous downregulation was not reproducible. Downregulation of *apvhal26* was observed in the active treatment group (*n* = 40) compared to the ds*GFP* control group (*n* = 40), but did not reach statistical significance. *Ago2* expression showed a significant increase compared to the control, although the high variability in the dataset limits the conclusiveness of this result. *Dicer1* expression remained significantly elevated in ds*Apvhal26*‐treated aphids relative to the control.

In none of the topical dsRNA delivery experiments targeting the secondary target gene *MIF1*, a significant downregulation of the target gene was achieved (Supporting Information S1: Figure 7).

Across both oral and topical delivery experiments, expression of *ago2* and *dicer1* was frequently elevated following dsRNA exposure, whereas reproducible target gene knockdown was variable between experimental repetitions.

### 3.3 Comparative Analysis of Gene Expression After dsRNA/Formulation Treatment

#### 3.3.1 Gene Expression Following Oral Delivery of Formulated Dsrna

**Figure 8:**
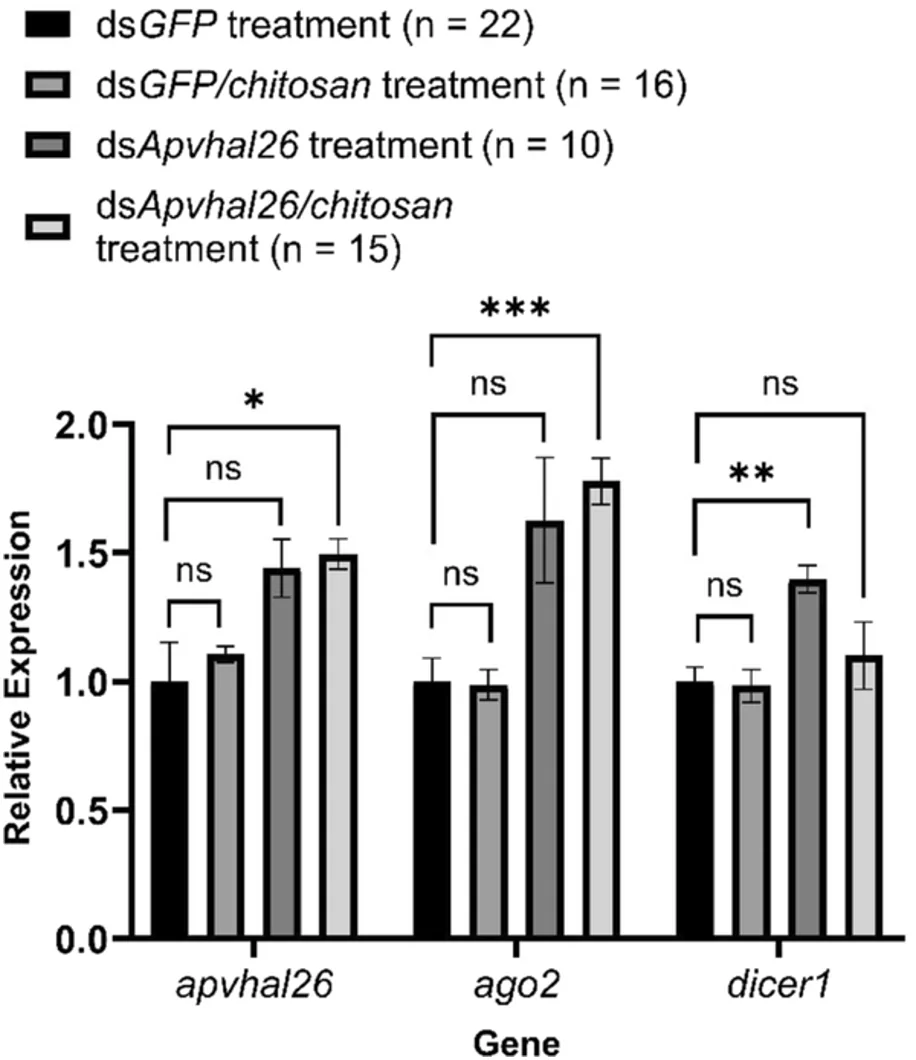
Relative gene expression following feeding with ds*Apvhal26*/chitosan. Relative expression of *apvhal26*, *ago2*, and *dicer1* in *Acyrthosiphon pisum* following oral delivery of dsRNA formulated with chitosan nanoparticles. Aphids were fed an artificial diet containing nuclease‐free water (H_2_O), control ds*GFP*, unformulated ds*Apvhal26*, or ds*Apvhal26* complexed with chitosan nanoparticles (dsRNA/chitosan mass ratio 1:4) for 72 h. Expression levels were normalized to the reference gene *SDHB* and are shown relative to the H_2_O control. No significant reduction in target gene *apvhal26* expression was observed. *Ago2* transcript abundance was significantly elevated in both ds*Apvhal26* and ds*Apvhal26*/chitosan treatment groups, with the strongest increase observed following chitosan formulation. *Dicer1* expression increased significantly only in the unformulated ds*Apvhal26* treatment, but not in the other groups. Bars represent mean ± SEM. Asterisks denote statistically significant differences compared to the control (“ns” not significant; *p* < 0.05; *p < 0.01; **p < 0.001, unpaired *t* test).

Oral delivery of chitosan‐formulated dsRNA altered the expression of RNAi‐associated genes but did not result in detectable target gene knockdown (Figure 8). Expression of *apvhal26* did not decrease following treatment with ds*Apvhal26*/chitosan. Instead, transcript abundance remained comparable to the control treatment, with a modest but significant increase observed in the ds*Apvhal26*/chitosan group relative to ds*GFP*. *Ago2* transcript abundance was significantly elevated in both ds*Apvhal26* and ds*Apvhal26*/chitosan treatment groups, with the strongest increase observed following chitosan formulation. *Dicer1* expression increased significantly only in the unformulated ds*Apvhal26* treatment, but not in the other groups. Overall, chitosan formulation was associated with changes in the expression of RNAi‐associated genes but did not produce measurable suppression of the target gene under the conditions tested.

**Figure 9:**
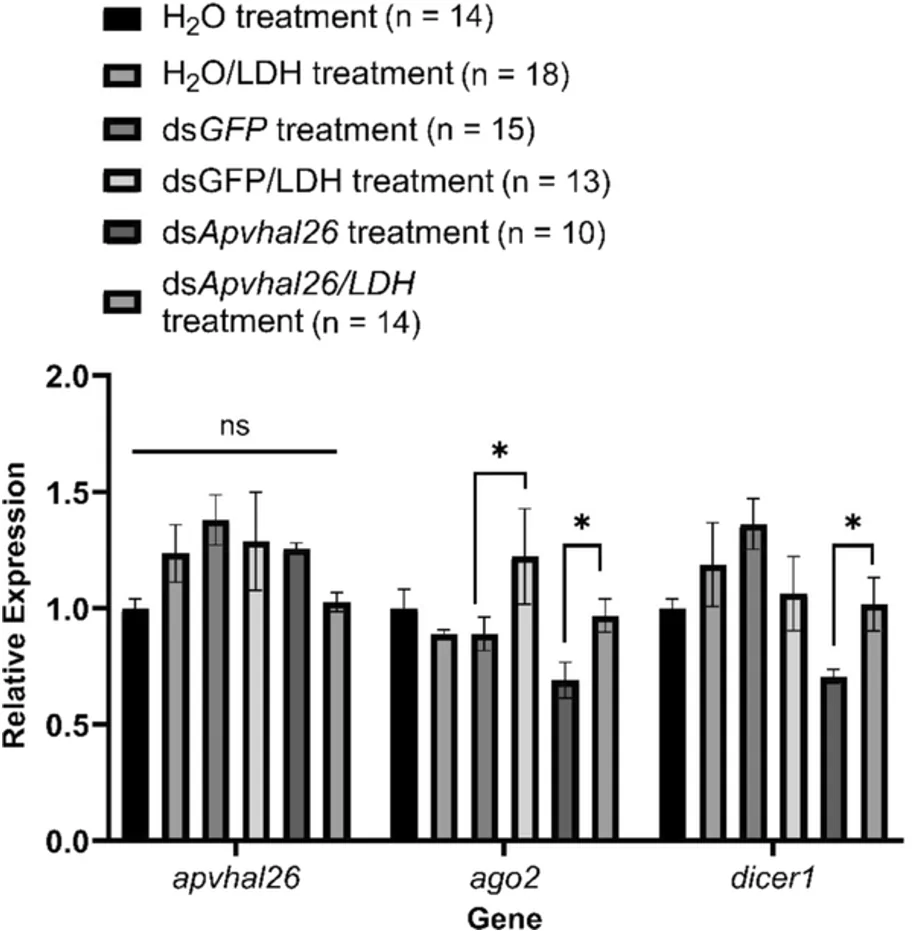
Relative gene expression following Feeding with ds*Apvhal26*/LDH. Relative expression of *apvhal26*, *ago2*, and *dicer1* in *Acyrthosiphon pisum* following oral delivery of dsRNA formulated with LDH nanoparticles. Aphids were fed an artificial diet containing nuclease‐free water (H_2_O), control ds*GFP*, unformulated ds*Apvhal26*, or ds*Apvhal26* bound to LDH nanosheets (dsRNA/LDH mass ratio 1:7) for 72 h. Expression levels were normalized to the reference gene *SDHB* and are shown relative to the H_2_O control. No significant reduction in *apvhal26* transcript abundance was detected in any treatment group. *Ago2* was significantly higher in aphids receiving ds*GFP/LDH* and ds*Apvhal26/LDH*, compared with their respective unformulated dsRNA treatments. *Dicer1* expression showed a significant difference only between ds*Apvhal26* and ds*Apvhal26*/chitosan. Bars represent mean ± SEM. Asterisks denote statistically significant differences compared to the control (“ns” not significant; *p* < 0.05; *p < 0.01; **p < 0.001).

Similar patterns were observed following oral delivery of LDH‐formulated dsRNA (dsRNA/LDH mass ratio of 1:7) (Figure 9). No significant reduction in *apvhal26* transcript abundance was detected in any treatment group. In contrast, *ago2* was significantly higher in aphids receiving ds*GFP/LDH* and ds*Apvhal26/LDH*, compared with their respective unformulated dsRNA treatments. *Dicer1* expression showed a similar tendency, although statistically significant differences were only observed between ds*Apvhal26* and ds*Apvhal26*/chitosan. Overall, LDH formulation was associated with increased expression of RNAi‐associated genes but did not result in detectable target gene silencing.

## 4 Discussion

The use of food dyes as ingestion markers confirmed successful feeding of *A. pisum* on the artificial diet in the feeding chambers, which evidenced distinct blue and red pigmentation. Brilliant Blue is trapped in the alimentary tract, whereas Allura Red AC appears to pass the stomach membrane and is probably metabolized, consistent with observations in other organisms (Aguilar et al. 2010). These results validate that the feeding setup enabled reliable dietary uptake through the stylet pathway and provide a simple, non‐invasive method to confirm exposure in ingestion‐dependent delivery experiments such as oral RNAi.

Feeding assays with Cy3‐labeled dsRNA provided further information on the distribution and persistence of fluorescently labeled dsRNA following oral uptake. Fluorescence was detectable in the aphid gut as early as 30 min post‐feeding and persisted for up to 120 h, while no comparable signal was observed in water‐fed controls. Fluorescence remained predominantly localized in the stomach and was not detected in the intestine and beyond, despite the small molecular size of the Cy3 fluorophore (approximately 616–631 Da, 1–2 nm), which should probably allow passage across the connection between the stomach and the intestine. This observation suggests either physical retention of Cy3‐labeled dsRNA conjugates in the stomach or persistence of the fluorescent Cy3 signal, probably binding non‐specifically to the stomach membrane, following dsRNA degradation through interaction with the perimicrovillar membrane. Importantly, fluorescence confirms ingestion but not the integrity of dsRNA. Nevertheless, the persistence and localization of the fluorescent signal align with earlier reports that systemic transport of dsRNA in hemipterans is inefficient (Jain et al. 2021; Nitnavare et al. 2021).

However, because in this study similar fluorescence was also observed in the Cy3‐only control treatment, the signal cannot be attributed exclusively to intact dsRNA‐Cy3 complexes. The observed fluorescence therefore demonstrates exposure to and persistence of Cy3‐associated material within the aphid gut, but does not by itself establish that intact dsRNA was retained or transported within the gut. The persistence of the signal may reflect retention of fluorescently labeled dsRNA, dissociation or degradation of the dsRNA with persistence of the Cy3 fluorophore, or association of fluorescent material with the stomach‐associated structures such as the perimicrovillar membrane. Consequently, the present imaging data provide evidence for the localization and persistence of fluorescent material following oral exposure, but cannot resolve the molecular form of the retained material.

These observations nevertheless provide a useful basis for comparison with previous studies of dsRNA uptake in hemipterans. Gurusamy et al. (2020) demonstrated that orally delivered Cy3‐labeled dsRNA in the southern green stink bug (SGSB) was internalized by midgut cells but failed to reach peripheral tissues such as the fat body or epidermis, whereas injected dsRNA distributed systemically. Notably, dsRNA, siRNA, and short hairpin RNA (shRNA) were all detected in midgut cells after feeding, yet were similarly restricted from trans‐epithelial transport, indicating that, apart from dsRNA size, other factors determine systemic dissemination. Together with the present observation, these findings are consistent with the possibility that oral delivery of naked dsRNA in hemipterans is limited, in part, by barriers to midgut‐to‐hemocoel transport rather than initial uptake, providing a plausible explanation for the frequent observation of elevated expression of RNAi‐associated genes in the current study without consistent target gene knockdown following feeding‐based delivery.

Ex vivo degradation assays further demonstrated the vulnerability of naked dsRNA to rapid enzymatic breakdown in aphid BE. Unformulated ds*Apvhal26* showed visible degradation after 1 h and was almost completely degraded after 5 h, consistent with strong nuclease activity reported in aphid hemolymph and gut tissues (Christiaens and Smagghe 2014; Peng et al. 2018; Allen and Walker 2012; Wynant et al. 2014). Although BE provided a practical approach for assessing nuclease‐mediated degradation within the aphid, gut‐specific extracts would provide more direct information regarding digestive nuclease activity and represent an important direction for future work.

Chitosan‐formulated dsRNA displayed markedly increased stability, with detectable signals persisting after extended incubation. This confirms that chitosan substantially delays enzymatic degradation of dsRNA in aphid BE and provides prolonged protection in a nuclease‐rich environment, thereby corroborating earlier studies suggesting that nanoparticle encapsulation can shield dsRNA from nuclease‐driven degradation, enhancing its functional lifetime in hostile biological environments (Zhang et al. 2010; Zhou et al. 2023). A small fraction of dsRNA was gradually released from chitosan complexes yet remained detectable despite nuclease exposure, consistent with reports from the Colorado potato beetle midgut lumen (Vatanparast and Amari 2025).

Our results are consistent with Cao et al. (2018), who demonstrated that RNAi responses in *A. pisum* are weak and transient compared to the highly RNAi‐susceptible beetle *Tribolium castaneum*, largely due to rapid dsRNA degradation in aphid saliva, gut extracts, and hemolymph. While dsRNA remained stable for days in beetle diets and body fluids, thereby enabling sustained gene knockdown and mortality, aphid‐associated dsRNA was degraded within minutes ex vivo and within 24–72 h during feeding. Our degradation assays directly corroborate these observations and further show that both chitosan and LDH formulations markedly improve dsRNA persistence, highlighting nuclease protection as an important factor influencing RNAi efficiency in hemipterans.

Functionally, oral delivery of dsRNA targeting *apvhal26* resulted in significant target gene transcript reduction in the initial feeding experiment, alongside notably increased *ago2* and *dicer1* expression, demonstrating that aphids transcriptionally respond to exposure to exogenous dsRNA. However, these results were not consistently reproducible, with subsequent experiments showing weaker or non‐significant knockdown. Such variability likely reflects sensitivity to multiple parameters, including aphid clonal background, dsRNA dosage and exposure duration, and sampling time relative to dsRNA ingestion. Notably, increased expression of *ago2* and *dicer1* was also observed in treatments lacking measurable target gene knockdown, suggesting that recognition of exogenous dsRNA and transcriptional induction of RNAi‐associated genes can occur independently of productive target gene silencing. Consequently, expression differences of these RNAi‐associated genes alone should not be interpreted as evidence of effective and specific RNAi pathway activation. Previous studies have similarly reported genotype‐ and developmental stage‐dependent RNAi responses in aphids, with short‐lived silencing effects that may be missed depending on the time point chosen for transcript analysis (Cao et al. 2018; Jain et al. 2021).

Similar variability was observed following topical dsRNA delivery. One experimental replicate produced significant *apvhal26* knockdown, whereas subsequent repetitions resulted in only modest or non‐significant responses despite upregulation of RNAi‐associated genes. Although topical application of dsAp*vhal26* elicited measurable responses, these data alone cannot determine whether dsRNA entered through the cuticle, intersegmental membranes, spiracles, or other external structures, as the precise route of uptake remains unresolved. Visualization of topically applied fluorescently labeled dsRNA using confocal microscopy would help identify the tissues involved in uptake and distinguish between potential entry routes. For *MIF1*, topical delivery failed to reduce transcript levels, suggesting that target gene localization and tissue accessibility strongly influence RNAi efficacy (Liu et al. 2021). Together with rapid dsRNA degradation and limited systemic spread, our data align with the broader consensus that, despite extensive investigation, RNAi remains unreliable in hemipterans, where gene silencing outcomes are often weak, transient, or highly variable, in contrast to the robust and systemic RNAi responses in coleopterans (Christiaens and Smagghe 2014; Jain et al. 2021; Cao et al. 2018).

Aphids fed with LDH‐formulated ds*Apvhal26* exhibited a stronger transcriptional response of RNAi‐associated genes than aphids receiving chitosan‐formulated dsRNA. Although no significant *apvhal26* downregulation was achieved, *ago2* expression showed a consistently significant increase relative to controls and unformulated dsRNA treatments. Several mutually non‐exclusive mechanisms may explain this observation. LDH may improve dsRNA stability and bioavailability, thereby enhancing recognition of exogenous dsRNA. Alternatively, nanoparticle carriers themselves can induce stress‐related transcriptional responses that overlap with RNAi‐associated pathways (Monica and Janarthanan 2026). In many organisms, both chitosan and LDH have been reported to interact with biological membranes and may induce stress‐ or defense‐related responses, potentially leading to transcriptional abundance of RNAi‐associated genes independent of effective target gene silencing (Chun and Chandrasekaran 2019; Jing et al. 2022). Consequently, increased expression of *ago2* and *dicer1* cannot be interpreted as unequivocal evidence of productive RNAi pathway activation. Similar observations have been reported across multiple insect systems, where enhanced dsRNA stability or uptake, often activated through nanoparticle formulations, leads to increased transcript abundance of RNAi‐associated genes without consistent target gene silencing, underscoring that induction of *ago2* and *dicer1* reflects dsRNA recognition rather than RNAi efficacy (Christiaens and Smagghe 2014; Cao et al. 2018; Zhu and Palli 2020; Cheng et al. 2024). However, it should be noted that direct feeding of LDH‐bound dsRNA represents an artificial exposure scenario, as under field conditions, LDH would be applied as a foliar spray, with aphid uptake depending on prior adsorption to the leaf surface, entry into plant tissues, and subsequent acquisition during phloem feeding. Although LDH nanosheets can facilitate dsRNA stability and transport within plant tissue (Cheng et al. 2024), the extent to which intact LDH‐dsRNA complexes are ingested by aphids under natural conditions remains uncertain. Moreover, the present data do not allow discrimination between RNAi‐specific activation and nanoparticle‐induced stress responses, as nanoparticle carriers can interact with cells and can influence physiological and molecular responses in insects; therefore, the increased expression of *ago2* and *dicer1* observed in the present study may not exclusively reflect enhanced RNAi activity (Shahzad and Manzoor 2021; Ortolá and Daròs 2024; Liu et al. 2025a).

This study clearly highlights several remaining challenges in the application of RNAi‐based products for hemipteran control but also provides future perspectives. While BE provides a practical measure of overall nuclease activity present in *A. pisum*, they do not distinguish the relative contribution of digestive and non‐digestive tissues. Cy3 imaging confirmed the oral uptake of dsRNA and verified feeding as a viable dsRNA delivery method. As topical application of Cy3‐labeled dsRNA followed by confocal fluorescence microscopy was beyond the scope of this study, the biological responses observed following topical application indicate that externally applied dsRNA can interact with aphids under the experimental conditions used. To further expand on these findings, visualization of topically applied fluorescently labeled dsRNA will help to clarify the uptake routes following cuticular application.

A persistent challenge throughout this study was the limited reproducibility of target gene knockdown. Despite evidence of dsRNA uptake, stabilization by nanoparticle formulations, and frequent induction of *ago2* and *dicer1* gene expression, reproducible target gene silencing remained inconsistent across experimental repetitions. These findings suggest that RNAi efficiency in aphids is constrained by multiple interacting biological barriers that may vary between experimental repetitions. Moreover, although increased *ago2* and *dicer1* expression demonstrate a transcriptional response to exogenous dsRNA, these markers cannot distinguish between productive RNAi pathway activity leading to target mRNA degradation and broader cellular responses to dsRNA or nanoparticle exposure. Additional mechanistic studies examining siRNA production, intracellular dsRNA trafficking, and protein‐level responses would further clarify the relationship between activation and effective gene silencing. Overall, our findings indicate that successful RNAi in aphids depends on the coordinated interaction of multiple biological processes, including dsRNA stability, uptake, intracellular processing, and target accessibility. Continued optimization of delivery systems, improved understanding of nanoparticle release dynamics under aphid gut conditions, and further mechanistic investigation will be essential to translate enhanced dsRNA stability and recognition into reliable and biologically meaningful RNAi responses in hemipteran pests.

## 5 Conclusion

This study investigated key barriers limiting RNAi efficiency in *A. pisum* by investigating dsRNA delivery, stability, and functional outcomes. While orally delivered dsRNA was reliably ingested, and nanoparticle formulations improved its persistence in a nuclease‐rich environment, consequent target gene silencing remained difficult to achieve despite increased transcript abundance of RNAi‐associated genes.

Ex vivo degradation assays revealed rapid breakdown of unformulated dsRNA in aphid BE, showing complete degradation within 2 h, identifying nuclease‐mediated degradation as a major biological barrier to RNAi. Formulation with chitosan nanoparticles significantly improved stability, delaying degradation and extending dsRNA persistence in a nuclease‐rich environment.

Functional assays targeting *apvhal26* and *MIF1* showed that gene knockdown was inconsistently achieved, whereas elevated expression of RNAi‐associated genes was observed more consistently. Upregulation of *ago2* and *dicer1* occurred in most treatments, indicating either recognition of dsRNA even in the absence of measurable target gene silencing or a stress‐related response to exogenous dsRNA. Similar variability was observed following topical dsRNA delivery. Rather than identifying a single limiting factor, our findings suggest that RNAi efficacy in aphids is constrained by multiple interacting biological barriers. Rapid dsRNA degradation, tissue accessibility, delivery route, and the disconnection between elevated expression of RNAi‐related genes and reproducible target gene silencing together illustrate the complexity of achieving reliable RNAi responses in aphids.

This underlines the importance of careful target gene selection, as genes with intracellular or gut‐associated functions may be more amenable to RNAi than those requiring systemic silencing of secreted proteins. Further, nanoparticle formulations influenced RNAi outcomes. Chitosan primarily improved dsRNA stability in the presence of aphid nucleases, whereas LDH formulations were associated with stronger expression of RNAi‐associated genes, suggesting that LDH formulations facilitate dsRNA recognition. However, neither approach consistently translated into robust gene silencing, emphasizing that improved dsRNA delivery alone is insufficient to overcome the multiple barriers limiting RNAi efficacy.

Together, these findings refine our understanding of the biological barriers that limit RNAi in aphids and provide a framework for improving future RNAi‐based strategies. Continued optimization of dsRNA formulations, delivery systems, and target selection, combined with a better understanding of cellular uptake and intracellular processing, will be essential for achieving reproducible gene silencing. Although significant challenges remain, the present study demonstrates that several of these barriers can be partially mitigated, supporting the continued development of RNAi as a species‐specific and environmentally sustainable approach for aphid management.

## Author Contributions

**Lisa Merkel:** investigation, writing – original draft, methodology, writing – review and editing, formal analysis. **Mohammad Vatanparast:** investigation, methodology, formal analysis, writing – review and editing. **Torsten Will:** conceptualization, funding acquisition, writing – review and editing, supervision. **Silvio Erler:** conceptualization, funding acquisition, writing – review and editing, supervision. **Khalid Amari:** conceptualization, investigation, funding acquisition, methodology, writing – review and editing, formal analysis, supervision.

## Conflicts of Interest

The authors declare no conflicts of interest.

## Supporting information


Supporting File

## Data Availability

The data that support the findings of this study are available on request from the corresponding author. The data are not publicly available due to privacy or ethical restrictions.

## References

[arch70210-bib-0001] Abbasi, E. , M. Vahedi , M. Bagheri , S. Gholizadeh , H. Alipour , and M. D. Moemenbellah‐Fard . 2022. “Monitoring of Synthetic Insecticides Resistance and Mechanisms Among Malaria Vector Mosquitoes in Iran: A Systematic Review.” Heliyon 8, no. 1: e08830. 10.1016/j.heliyon.2022.e08830. 35128113 PMC8808063

[arch70210-bib-0002] Abdellatef, E. , T. Will , A. Koch , J. Imani , A. Vilcinskas , and K. H. Kogel . 2015. “Silencing the Expression of the Salivary Sheath Protein Causes Transgenerational Feeding Suppression in the Aphid *Sitobion avenae* .” Plant Biotechnology Journal 13, no. 6: 849–857. 10.1111/pbi.12322. 25586210

[arch70210-bib-0003] Aguilar, F. , B. Dusemund , P. Galtier , et al. 2010. “Scientific Opinion on the Re‐Evaluation of Canthaxanthin (E 161 g) as a Food Additive: Re‐Evaluation of Canthaxanthin (E 161 g) as a Food Additive.” EFSA Journal 8, no. 10: 1852. 10.2903/j.efsa.2010.1852.

[arch70210-bib-0004] Akashe, M. M. , U. V. Pawade , and A. V. Nikam . 2018. “Classification of Pesticides: A Review.” International Journal of Research in Ayurveda and Pharmacy 9, no. 4: 144–150. 10.7897/2277-4343.094131.

[arch70210-bib-0005] Almasi, R. , and K. Amari . 2021. “Harnessing Environmental‐RNAi to Win an Enduring Battle: Plant Versus Pathogen.” Journal of Phytopathology 169, no. 11–12: 649–657. 10.1111/jph.13040.

[arch70210-bib-0059] Allen, M. L. , and W. B. Walker , 3rd. 2012. “Saliva of Lygus Lineolaris Digests Double Stranded Ribonucleic Acids.” J Insect Physiol 58, no. 3: 391–396. 10.1016/j.jinsphys.2011.12.014. 22226823

[arch70210-bib-0006] Cao, M. , J. A. Gatehouse , and E. C. Fitches . 2018. “A Systematic Study of RNAi Effects and dsRNA Stability in *Tribolium castaneum* and *Acyrthosiphon pisum*, Following Injection and Ingestion of Analogous dsRNAs.” International Journal of Molecular Sciences 19, no. 4: 1079. 10.3390/ijms19041079. 29617308 PMC5979293

[arch70210-bib-0007] Carletto, J. , T. Martin , F. Vanlerberghe‐Masutti , and T. Brévault . 2010. “Insecticide Resistance Traits Differ Among and Within Host Races in *Aphis gossypii* .” Pest Management Science 66, no. 3: 301–307. 10.1002/ps.1874. 19908228

[arch70210-bib-0008] Cheng, X. , Q. Zhou , J. Xiao , et al. 2024. “Nanoparticle Ldh Enhances RNAi Efficiency of dsRNA in Piercing‐Sucking Pests by Promoting dsRNA Stability and Transport in Plants.” Journal of Nanobiotechnology 22, no. 1: 544. 10.1186/s12951-024-02819-4. 39237945 PMC11378424

[arch70210-bib-0009] Cherqui, A. , and W. F. Tjallingii . 2000. “Salivary Proteins of Aphids, a Pilot Study on Identification, Separation and Immunolocalisation.” Journal of Insect Physiology 46, no. 8: 1177–1186. 10.1016/S0022-1910(00)00037-8. 10818245

[arch70210-bib-0010] Christiaens, O. , and G. Smagghe . 2014. “The Challenge of RNAi‐Mediated Control of Hemipterans.” Current Opinion in Insect Science 6: 15–21. 10.1016/j.cois.2014.09.012. 32846663

[arch70210-bib-0011] Christiaens, O. , J. Niu , and C. Nji Tizi Taning . 2020. “Rnai in Insects: A Revolution in Fundamental Research and Pest Control Applications.” Insects 11, no. 7: 415. 10.3390/insects11070415. 32635402 PMC7411770

[arch70210-bib-0012] Chun, S. C. , and M. Chandrasekaran . 2019. “Chitosan and Chitosan Nanoparticles Induced Expression of Pathogenesis‐Related Proteins Genes Enhances Biotic Stress Tolerance in Tomato.” International Journal of Biological Macromolecules 125: 948–954. 10.1016/j.ijbiomac.2018.12.167. 30576730

[arch70210-bib-0013] Cristofoletti, P. T. , A. F. Ribeiro , C. Deraison , Y. Rahbé , and W. R. Terra . 2003. “Midgut Adaptation and Digestive Enzyme Distribution in a Phloem Feeding Insect, the Pea Aphid *Acyrthosiphon pisum* .” Journal of Insect Physiology 49, no. 1: 11–24. 10.1016/S0022-1910(02)00222-6. 12770012

[arch70210-bib-0014] De Rouck, S. , E. İnak , W. Dermauw , and T. van Leeuwen . 2023. “A Review of the Molecular Mechanisms of Acaricide Resistance in Mites and Ticks.” Insect Biochemistry and Molecular Biology 159: 103981. 10.1016/j.ibmb.2023.103981. 37391089

[arch70210-bib-0015] Dedryver, C. A. , A. Le Ralec , and F. Fabre . 2010. “The Conflicting Relationships Between Aphids and Men: A Review of Aphid Damage and Control Strategies.” Comptes Rendus Biologies 333, no. 6–7: 539–553. 10.1016/j.crvi.2010.03.009. 20541165

[arch70210-bib-0016] Dixon, A. F. G. , P. Kindlmann , J. Leps , and J. Holman . 1987. “Why There Are So Few Species of Aphids, Especially in the Tropics.” American Naturalist 129, no. 4: 580–592. 10.1086/284659.

[arch70210-bib-0017] Dong, H. , M. Chen , S. Rahman , H. S. Parekh , H. M. Cooper , and Z. P. Xu . 2014. “Engineering Small MgAl‐Layered Double Hydroxide Nanoparticles for Enhanced Gene Delivery.” Applied Clay Science 100: 66–75. 10.1016/j.clay.2014.04.028.

[arch70210-bib-0018] Ehrenberg, S. , O. Lewkowski , and S. Erler . 2019. “Dyeing but not Dying: Colourful Dyes as a Non‐Lethal Method of Food Labelling for in Vitro‐Reared Honey Bee (*Apis mellifera*) Larvae.” Journal of Insect Physiology 113: 1–8. 10.1016/j.jinsphys.2018.12.008. 30582906

[arch70210-bib-0019] Elston, K. M. , G. P. Maeda , J. Perreau , and J. E. Barrick . 2023. “Addressing the Challenges of Symbiont‐Mediated RNAi in Aphids.” PeerJ 11: e14961. 10.7717/peerj.14961. 36874963 PMC9983426

[arch70210-bib-0020] Fire, A. , S. Xu , M. K. Montgomery , S. A. Kostas , S. E. Driver , and C. C. Mello . 1998. “Potent and Specific Genetic Interference by Double‐Stranded RNA in *Caenorhabditis elegans* .” Nature 391, no. 6669: 806–811. 10.1038/35888. 9486653

[arch70210-bib-0021] Forgac, M. 2007. “Vacuolar ATPases: Rotary Proton Pumps in Physiology and Pathophysiology.” Nature Reviews Molecular Cell Biology 8, no. 11: 917–929. 10.1038/nrm2272. 17912264

[arch70210-bib-0022] Ghodke, A. B. , R. T. Good , J. F. Golz , D. A. Russell , O. Edwards , and C. Robin . 2019. “Extracellular Endonucleases in the Midgut of *Myzus persicae* May Limit the Efficacy of Orally Delivered RNAi.” Scientific Reports 9, no. 1: 11898. 10.1038/s41598-019-47357-4. 31417162 PMC6695413

[arch70210-bib-0062] Gurusamy, D. , J. L. Howell , S. C. R. R. Chereddy , J. Koo , and S. R. Palli . 2020. “Transport of orally delivered dsRNA in southern green stink bug, Nezara viridula.” In Archives of Insect Biochemistry and Physiology 104, no. 4, e21692. 10.1002/arch.21692. 32441400

[arch70210-bib-0023] Jain, R. G. , S. J. Fletcher , N. Manzie , et al. 2022. “Foliar Application of Clay‐Delivered RNA Interference for Whitefly Control.” Nature Plants 8, no. 5: 535–548. 10.1038/s41477-022-01152-8. 35577960

[arch70210-bib-0024] Jain, R. G. , K. E. Robinson , S. Asgari , and N. Mitter . 2021. “Current Scenario of RNAi‐Based Hemipteran Control.” Pest Management Science 77, no. 5: 2188–2196. 10.1002/ps.6153. 33099867

[arch70210-bib-0025] Jing, G. , L. Yang , H. Wang , J. Niu , Y. Li , and S. Wang . 2022. “Interference of Layered Double Hydroxide Nanoparticles With Pathways for Biomedical Applications.” Advanced Drug Delivery Reviews 188: 114451. 10.1016/j.addr.2022.114451. 35843506

[arch70210-bib-0026] Kolge, H. , K. Kadam , S. Galande , V. Lanjekar , and V. Ghormade . 2021. “New Frontiers in Pest Control: Chitosan Nanoparticles‐Shielded dsRNA as an Effective Topical RNAi Spray for Gram Podborer Biocontrol.” ACS Applied Bio Materials 4, no. 6: 5145–5157. 10.1021/acsabm.1c00349. 35006998

[arch70210-bib-0027] Liu, J. , Q. He , X. Lin , and G. Smagghe . 2025a. “Recent Progress in Nanoparticle‐Mediated RNA Interference in Insects: Unveiling New Frontiers in Pest Control.” Journal of Insect Physiology 167: 104884. 10.1016/j.jinsphys.2025.104884. 40939831

[arch70210-bib-0028] Liu, S. , M. Jaouannet , D. A. Dempsey , J. Imani , C. Coustau , and K. H. Kogel . 2020. “RNA‐Based Technologies for Insect Control in Plant Production.” Biotechnology Advances 39: 107463. 10.1016/j.biotechadv.2019.107463. 31678220

[arch70210-bib-0029] Liu, S. , M. J. Ladera‐Carmona , M. M. Poranen , A. J. E. van Bel , K. H. Kogel , and J. Imani . 2021. “Evaluation of dsRNA Delivery Methods for Targeting Macrophage Migration Inhibitory Factor MIF in RNAi‐Based Aphid Control.” Journal of Plant Diseases and Protection 128, no. 5: 1201–1212. 10.1007/s41348-021-00464-9.

[arch70210-bib-0030] Liu, Y. , J. Zhang , S. Li , et al. 2025b. “Chitosan Nanoparticle‐Mediated Delivery of dsRNA for Enhancing RNAi Efficiency in *Locusta migratoria* .” Pest Management Science 81, no. 9: 5260–5269. 10.1002/ps.8880. 40342233

[arch70210-bib-0031] Livak, K. J. , and T. D. Schmittgen . 2001. “Analysis of Relative Gene Expression Data Using Real‐Time Quantitative PCR and the 2^−ΔΔC^T Method.” Methods 25, no. 4: 402–408. 10.1006/meth.2001.1262. 11846609

[arch70210-bib-0032] Mitter, N. , E. A. Worrall , K. E. Robinson , et al. 2017a. “Clay Nanosheets for Topical Delivery of RNAi for Sustained Protection Against Plant Viruses.” Nature Plants 3, no. 2: 16207. 10.1038/nplants.2016.207. 28067898

[arch70210-bib-0033] Mitter, N. , E. A. Worrall , K. E. Robinson , Z. P. Xu , and B. J. Carroll . 2017b. “Induction of Virus Resistance by Exogenous Application of Double‐Stranded RNA.” Current Opinion in Virology 26: 49–55. 10.1016/j.coviro.2017.07.009. 28778033

[arch70210-bib-0034] Mitter, T. E. , and R. H. Dadd . 1964. “An Improved Method for Feeding Aphids on Artificial Diets.” Annals of the Entomological Society of America 57, no. 1: 139–140. 10.1093/aesa/57.1.139a.

[arch70210-bib-0035] Monica, K. , and S. Janarthanan . 2026. “Insects as Experimental Models to Study the Toxicological Effects of Nanoparticles: Concerns at the Behavioral, Physiological, Cellular, and Molecular Levels.” Journal of Applied Toxicology 46, no. 2: 419–433. 10.1002/jat.4960. 41047552

[arch70210-bib-0036] Mutti, N. S. , J. Louis , L. K. Pappan , et al. 2008. “A Protein From the Salivary Glands of the Pea Aphid, *Acyrthosiphon pisum*, Is Essential in Feeding on a Host Plant.” Proceedings of the National Academy of Sciences of the United States of America 105, no. 29: 9965–9969. 10.1073/pnas.0708958105. 18621720 PMC2481341

[arch70210-bib-0037] Naessens, E. , G. Dubreuil , P. Giordanengo , et al. 2015. “A Secreted MIF Cytokine Enables Aphid Feeding and Represses Plant Immune Responses.” Current Biology 25, no. 14: 1898–1903. 10.1016/j.cub.2015.05.047. 26119751

[arch70210-bib-0038] Nitnavare, R. B. , J. Bhattacharya , S. Singh , A. Kour , M. J. Hawkesford , and N. Arora . 2021. “Next Generation dsRNA‐Based Insect Control: Success So Far and Challenges.” Frontiers in Plant Science 12: 673576. 10.3389/fpls.2021.673576. 34733295 PMC8558349

[arch70210-bib-0039] Niu, J. , W. J. Yang , Y. Tian , et al. 2019. “Topical dsRNA Delivery Induces Gene Silencing and Mortality in the Pea Aphid.” Pest Management Science 75, no. 11: 2873–2881. 10.1002/ps.5457. 31038279

[arch70210-bib-0040] Omkar , ed. 2020. Sucking Pests of Crops. Springer Singapore.

[arch70210-bib-0041] Ortolá, B. , and J. A. Daròs . 2024. “RNA Interference in Insects: From a Natural Mechanism of Gene Expression Regulation to a Biotechnological Crop Protection Promise.” Biology 13, no. 3: 137. 10.3390/biology13030137. 38534407 PMC10968014

[arch70210-bib-0042] Palli, S. R. 2023. “RNAi Turns 25: Contributions and Challenges in Insect Science.” Frontiers in Insect Science 3: 1209478. 10.3389/finsc.2023.1209478. 38469536 PMC10926446

[arch70210-bib-0043] Pallis, S. , A. Alyokhin , B. Manley , T. Rodrigues , E. Barnes , and K. Narva . 2023. “Effects of Low Doses of a Novel dsRNA‐Based Biopesticide (Calantha) on the Colorado Potato Beetle.” Journal of Economic Entomology 116, no. 2: 456–461. 10.1093/jee/toad034. 36895198

[arch70210-bib-0060] Peng, Y. , K. Wang , W. Fu , C. Sheng , and Z. Han . 2018. “Biochemical Comparison of dsRNA Degrading Nucleases in Four Different Insects.” Front Physiol 9: 624. 10.3389/fphys.2018.00624. 29892232 PMC5985623

[arch70210-bib-0044] Pitino, M. , and S. A. Hogenhout . 2013. “Aphid Protein Effectors Promote Aphid Colonization in a Plant Species‐Specific Manner.” Molecular Plant‐Microbe Interactions 26, no. 1: 130–139. 10.1094/MPMI-07-12-0172-FI. 23035913

[arch70210-bib-0045] Pitino, M. , A. D. Coleman , M. E. Maffei , C. J. Ridout , and S. A. Hogenhout . 2011. “Silencing of Aphid Genes by dsRNA Feeding From Plants.” PLoS One 6, no. 10: e25709. 10.1371/journal.pone.0025709. 21998682 PMC3187792

[arch70210-bib-0046] Price, D. R. G. , and J. A. Gatehouse . 2008. “RNAi‐Mediated Crop Protection Against Insects.” Trends in Biotechnology 26, no. 7: 393–400. 10.1016/j.tibtech.2008.04.004. 18501983

[arch70210-bib-0047] Rodrigues, T. B. , S. K. Mishra , K. Sridharan , et al. 2021. “First Sprayable Double‐Stranded RNA‐Based Biopesticide Product Targets Proteasome Subunit Beta Type‐5 in Colorado Potato Beetle (*Leptinotarsa decemlineata*).” Frontiers in Plant Science 12: 728652. 10.3389/fpls.2021.728652. 34887882 PMC8650841

[arch70210-bib-0048] Shahzad, K. , and F. Manzoor . 2021. “Nanoformulations and Their Mode of Action in Insects: A Review of Biological Interactions.” Drug and Chemical Toxicology 44, no. 1: 1–11. 10.1080/01480545.2018.1525393. 30760084

[arch70210-bib-0049] Vatanparast, M. , and K. Amari . 2025. “Development and Efficacy of dsRNA Pesticides Targeting the Colorado Potato Beetle With Enhanced Stability via Chitosan Formulations.” Pesticide Biochemistry and Physiology 214: 106606. 10.1016/j.pestbp.2025.106606. 40915798

[arch70210-bib-0050] Vatanparast, M. , L. Merkel , and K. Amari . 2024. “Exogenous Application of dsRNA in Plant Protection: Efficiency, Safety Concerns and Risk Assessment.” International Journal of Molecular Sciences 25, no. 12: 6530. 10.3390/ijms25126530. 38928236 PMC11204322

[arch70210-bib-0051] Vellichirammal, N. N. , P. Gupta , T. A. Hall , and J. A. Brisson . 2017. “Ecdysone Signaling Underlies the Pea Aphid Transgenerational Wing Polyphenism.” Proceedings of the National Academy of Sciences of the United States of America 114, no. 6: 1419–1423. 10.1073/pnas.1617640114. 28115695 PMC5307454

[arch70210-bib-0052] Will, T. , A. Hewer , and A. J. E. van Bel . 2008. “A Novel Perfusion System Shows That Aphid Feeding Behaviour Is Altered by Decrease of Sieve‐Tube Pressure.” Entomologia Experimentalis et Applicata 127, no. 3: 237–245. 10.1111/j.1570-7458.2008.00687.x.

[arch70210-bib-0061] Wynant, N. , D. Santos , and R. Verdonck . 2014. Identification, Functional Characterization and Phylogenetic Analysis of Double Stranded RNA Degrading Enzymes Present in the gut of the Desert Locust, Schistocerca Gregaria.” Insect Biochem Mol Biol 46: 1–8. 10.1016/j.ibmb.2013.12.008. 24418314

[arch70210-bib-0053] Xu, Z. P. , G. S. Stevenson , C. Q. Lu , G. Q. Lu , P. F. Bartlett , and P. P. Gray . 2006. “Stable Suspension of Layered Double Hydroxide Nanoparticles in Aqueous Solution.” Journal of the American Chemical Society 128, no. 1: 36–37. 10.1021/ja056652a. 16390109

[arch70210-bib-0054] Yang, C. , H. Pan , Y. Liu , and X. Zhou . 2014. “Selection of Reference Genes for Expression Analysis Using Quantitative Real‐Time PCR in the Pea Aphid, *Acyrthosiphon pisum* (Harris) (Hemiptera, Aphidiae).” PLoS One 9, no. 11: e110454. 10.1371/journal.pone.0110454. 25423476 PMC4244036

[arch70210-bib-0055] Yang, W. , B. Wang , G. Lei , G. Chen , and D. Liu . 2022. “Advances in Nanocarriers to Improve the Stability of dsRNA in the Environment.” Frontiers in Bioengineering and Biotechnology 10: 974646. 10.3389/fbioe.2022.974646. 36051593 PMC9424858

[arch70210-bib-0056] Zhang, X. , J. Zhang , and K. Y. Zhu . 2010. “Chitosan/Double‐Stranded RNA Nanoparticle‐Mediated RNA Interference to Silence Chitin Synthase Genes Through Larval Feeding in the African Malaria Mosquito (*Anopheles gambiae*).” Insect Molecular Biology 19, no. 5: 683–693. 10.1111/j.1365-2583.2010.01029.x. 20629775

[arch70210-bib-0057] Zhou, H. , F. Wan , Y. Jian , et al. 2023. “Chitosan/dsRNA Polyplex Nanoparticles Advance Environmental RNA Interference Efficiency Through Activating Clathrin‐Dependent Endocytosis.” International Journal of Biological Macromolecules 253, no. Pt 4: 127021. 10.1016/j.ijbiomac.2023.127021. 37741481

[arch70210-bib-0058] Zhu, K. Y. , and S. R. Palli . 2020. “Mechanisms, Applications, and Challenges of Insect RNA Interference.” Annual Review of Entomology 65: 293–311. 10.1146/annurev-ento-011019-025224. PMC9939233 31610134

