# Supplementary material for: Delivery, Persistence, and Functional Response of dsRNA in Acyrthosiphon pisum: Progress and Remaining Barriers
Source: Arch Insect Biochem Physiol. 2026 Sep 14;123(1):e70210. doi: 10.1002/arch.70210 (PMC13575441; doi:10.1002/arch.70210)
Supplement: Supplementary file 1 [file ARCH-123-e70210-s001.docx]

**Supplementary Material**

**Preparation of Chitosan and LDH nanoparticles**

1. **Chitosan**

Chitosan/dsRNA nanoparticles were prepared using chitosan derived from crab shells (catalog no. C3646-25G, ≥75% deacetylated; Sigma-Aldrich, Milwaukee, WI, USA). Chitosan was dissolved in sodium acetate buffer (0.1 M sodium acetate-0.1 M acetic acid, pH 4.5) to yield a 0.02% (*w/v*) working solution.

For dsRNA/chitosan-nanoparticle formation, 32 μg of dsRNA suspended in 100 μL of 50 mM sodium sulfate was added to 100 μL of the chitosan working solution. The quantities of dsRNA and chitosan were adjusted to ensure optimal electrostatic interaction and nanoparticle assembly. To determine the ideal dsRNA/chitosan loading ratio, *in vitro* transcribed ds*GFP* and ds*Apvhal26* (500 ng) were each tested at ratios ranging from 1:0 to 1:10 (*w/w*). Following mixing, the solutions were heated to 55 °C for 1 minute and immediately vortexed at high speed for 30 seconds using a vortex mixer (IKA Minishaker MS1, IKA-Werke GmbH & Co. KG, Staufen, Germany), initiating nanoparticle complexation. The resulting suspension was centrifuged at 13.000 × g for 10 min at room temperature to pellet the nanoparticles. The supernatant was discarded, and the pellet was washed with 100 μL of nuclease-free water. After a second centrifugation step under the same conditions, the pellet was resuspended in 100 μL of nuclease-free water.

To ensure even dispersion, the nanoparticle suspension was briefly sonicated using an ultrasonic probe (Branson SFX 250 Digital Sonifier®, Emerson Electric Co., St. Louis, MO, USA) at 10% amplitude and 20 kHz for 5–10 sec. Complete dsRNA incorporation was confirmed by a 1% agarose gel electrophoresis assay. For both ds*GFP* and ds*Apvhal26*, optimal complexation was achieved at a chitosan/dsRNA mass ratio of 4:1 (Fig. S1).

2. **LDH**

MgAl-layered double hydroxide (MgAl-LDH) nanoparticles were synthesized following a modified protocol adapted from previous studies (Dong et al. 2014; Mitter et al. 2017a; Xu et al. 2006). On Day 1, a methanolic solution (10 mL) containing 6 mmol of magnesium nitrate hexahydrate (Mg(NO₃)₂·6H₂O, ≥99.0%; 1.54 g) and 2 mmol of aluminum nitrate nonahydrate (Al(NO₃)₃·9H₂O, ≥98.0%; 0.75 g) was prepared. This precursor solution was added dropwise under vigorous stirring to a separate methanolic solution of sodium hydroxide (NaOH, ≥97.0%, pellets; 0.64 g, 16 mmol in 40 mL methanol). The reaction mixture was stirred for 30 min to allow for initial precipitation. The resulting slurry was collected by centrifugation at 1700 × g for 20 min and washed twice with fresh methanol (40 mL each). The washed precipitate was then transferred to a Teflon^®^-lined autoclave and heated at 80 °C for 18 h.

On day 2, the autoclave was allowed to cool down to room temperature. The precipitate was collected by centrifugation at 1700 × g for 20 min, washed twice with deionized water, and left at room temperature with occasional hand agitation. After 4-6 days, the dispersion became transparent, indicating successful formation of a homogeneous suspension of MgAl-LDH nanoparticles. To confirm the correct concentration, 500 µL aliquots of the LDH solution were dried in triplicate, and the dried material was then weighed. The final concentration of LDH in suspension was determined to be approximately 6-7 mg/mL.

To determine the optimal loading ratio for ds*GFP* and ds*Apvhal26* binding onto MgAl-LDH nanoparticles, both dsRNAs and LDH were prepared from 1 μg/μL stock solutions. Loading reactions were set up using dsRNA/LDH mass ratios ranging from 1:0 to 1:10. For each condition, 1 μL (1 μg) of dsRNA was mixed with 0-10 μL (0-10 μg) of LDH, and nuclease-free water was added to bring the total reaction volume to 11 μL. Samples were incubated at room temperature for 30 minutes to allow electrostatic complexation of dsRNA with LDH nanosheets. During incubation, each reaction mixture was briefly vortexed every 5 min to maintain homogeneity. Following incubation, loading efficiency was assessed by 1% agarose gel electrophoresis. The absence of free dsRNA migration in the gel confirmed complete loading. For both ds*GFP* and ds*Apvhal26*, optimal complexation was achieved at a dsRNA/LDH mass ratio of 1:7 (Fig. S2).

**Figure S1. The amounts of dsRNA and chitosan should be balanced to achieve efficient electrostatic interactions between them.** Complete loading was achieved at a chitosan/ds*GFP* mass ratio of 4:1 (**a.**), and a ds*Apvhal26*/chitosan mass ratio of 4:1 (**b.**). 'M' indicates the DNA size marker (1 kb ladder).

**Figure S2. The amounts of dsRNA and LDH should be balanced for efficient electrostatic interactions between LDH and dsRNA.** Complete loading was achieved at a ds*GFP*/LDH mass ratio of 1:7 (**a.**), and ds*Apvhal26*/LDH mass ratio of 1:7 (**b.**). 'M' indicates the DNA size marker (1 kb ladder).

**Figure S3. Mean total protein concentration determined by BCA protein assay.** Standard curve generated using bovine serum albumin (BSA) as a reference to determine total protein concentration in *A. pisum* whole-body extract.

| **a. ds*GFP*+Cy3 Feeding Bioassay** | | | |
| --- | --- | --- | --- |
| Time (h) post-feeding | Bright Field | | Fluorescence |
| 0.5 |  | |  |
| 1 |  | |  |
| 2 |  | |  |
| 24 |  | |  |
| 48 |  | |  |
| 96 |  | |  |
| 120 |  | |  |
|  | | | |
| **b. Cy3-Labelling Agent Feeding Bioassay** | | | |
| Time (h) post-feeding | Bright Field | | Fluorescence |
| 0.5 |   500 µm | |   500 µm |
| 1 |   500 µm  500 µm | |  |
| 2 |   500 µm  500 µm | |  |
| 24 |   500 µm  500 µm | |  |
| 48 |   500 µm  500 µm | |  |
| 96 |  | |  |
| 120 |  | |   500 µm  500 µm |
|  | | | |
| **c. H_2_O Control Feeding Bioassay** | | | |
| Time (h) post Feeding | Bright Light | Fluorescence | |
| 0.5 |  |  | |

500 µm

500 µm

**Figure S4. Uptake of fluorescent-labeled dsRNA from artificial diet.** The artificial diet contained Cy3-labeled ds*GFP* (5 μg). Pictures were taken at 0.5, 1, 2, 24, 48, 96, and 120 h after feeding and show the fluorescence signal and its localization in *A. pisum*. Fluorescence was detected inside the insect gut. Left panels: stereo-microscopic analysis under bright field; right panels: fluorescence stereo-microscopic analysis: excitation/emission wavelength (494 nm/515 nm), scale bars = 500 μm.

**Figure S5. Relative expression of MIF1, ago2, and dicer1 in A. pisum after dsMIF1 feeding bioassay**. Quantification of MIF1, ago2, and dicer1 transcripts in A. pisum by qRT-PCR. The MIF1 mRNA levels were significantly lower in aphids fed on dsMIF1. Gene expression of argonaute2 was significantly overexpressed in aphids feeding on dsMIF1 compared to H_2_O. Gene expression of dicer1 also displayed significant overexpression in aphids feeding on dsMIF1 compared to the control group H_2_O. (* p ≤ 0.05; ** p ≤ 0.01; *** p ≤ 0.001 ns = not significant, unpaired t-test).

**Figure S6. Inconsistent results of relative expression of *MIF1*, *ago2*, and *dicer1* in *A. pisum* after ds*MIF1* feeding**. The *MIF1,* *ago2*, and *dicer1* mRNA levels display no significant differences in aphids feeding on any treatment.

**Figure S7. Inconsistent results of relative expression of *MIF1* in *A. pisum* after topical dsRNA delivery**. No lower *MIF1* mRNA transcript levels in aphids treated with ds*MIF1* were detected, but even significant overexpression compared to H_2_O-treated aphids (** *p* ≤ 0.01, unpaired t-test). Also ds*GFP*-treated aphids differed from the water control (* *p* ≤ 0.05, unpaired t-test).

| **Gene** | **Primer direction** | **Sequence (5′ → 3′)** | **Reference** | |
| --- | --- | --- | --- | --- |
| *V-ATPase* | Forward | GAGGAAATCGACGCCAAAGC | | (Niu et al. 2019) |
|  | Reverse | CTTGAGTCGCTGGTGTTGGA | |  |
| *MIF1* | Forward | TGCAGCTGTCTTGTATCCTCT | | This study |
|  | Reverse | ACATTGGATGAACTTTGGTCCG | |  |
| *SDHB* (reference gene) | Forward | CTGAATTCCTGTGGACCTATGG | | (Yang et al. 2014) |
|  | Reverse | ACGGCAAGAACGCCTAAA | |  |
| *Dicer1* | Forward | CTGACAAGGCAGCAGATAAAGG | | (Elston et al. 2023) |
|  | Reverse | GCGGCAACCTTAGCTTCTTT | |  |
| *Argonaute2* | Forward | TCCCGACCAACCAAGTATCA | | (Elston et al. 2023) |
|  | Reverse | GCCGGATATGAAACAGAACGAG | |  |

**Table S1.** Primer sequences for qRT-PCR analysis to determine target gene transcription levels in RNA extracted from dsRNA-treated *A. pisum*.
